# Evaluating next‐generation sequencing (NGS) methods for routine monitoring of wild bees: Metabarcoding, mitogenomics or NGS barcoding

**DOI:** 10.1111/1755-0998.13013

**Published:** 2019-04-29

**Authors:** Morgan Gueuning, Dominik Ganser, Simon Blaser, Matthias Albrecht, Eva Knop, Christophe Praz, Juerg E. Frey

**Affiliations:** ^1^ Research Group Molecular Diagnostics, Genomics and Bioinformatics Agroscope Wädenswil Switzerland; ^2^ Institute of Biology University of Neuchatel Neuchatel Switzerland; ^3^ Institute of Ecology and Evolution University of Bern Bern Switzerland; ^4^ Agroecology and Environment Agroscope Zürich Switzerland; ^5^ Department of Epidemiology and Public Health, Swiss Tropical and Public Health Institute University of Basel Basel Switzerland

**Keywords:** conservation biology, DNA barcoding, insects, molecular identification, pollinators, survey

## Abstract

Implementing cost‐effective monitoring programs for wild bees remains challenging due to the high costs of sampling and specimen identification. To reduce costs, next‐generation sequencing (NGS)‐based methods have lately been suggested as alternatives to morphology‐based identifications. To provide a comprehensive presentation of the advantages and weaknesses of different NGS‐based identification methods, we assessed three of the most promising ones, namely metabarcoding, mitogenomics and NGS barcoding. Using a regular monitoring data set (723 specimens identified using morphology), we found that NGS barcoding performed best for both species presence/absence and abundance data, producing only few false positives (3.4%) and no false negatives. In contrast, the proportion of false positives and false negatives was higher using metabarcoding and mitogenomics. Although strong correlations were found between biomass and read numbers, abundance estimates significantly skewed the communities' composition in these two techniques. NGS barcoding recovered the same ecological patterns as morphology. Ecological conclusions based on metabarcoding and mitogenomics were similar to those based on morphology when using presence/absence data, but different when using abundance data. In terms of workload and cost, we show that metabarcoding and NGS barcoding can compete with morphology, but not mitogenomics which was consistently more expensive. Based on these results, we advocate that NGS barcoding is currently the seemliest NGS method for monitoring of wild bees. Furthermore, this method has the advantage of potentially linking DNA sequences with preserved voucher specimens, which enable morphological re‐examination and will thus produce verifiable records which can be fed into faunistic databases.

## 1 INTRODUCTION

During the last decades, insect pollinators, and especially bees, have declined in several regions of the world (Bartomeus, Stavert, Ward, & Aguado, 2019; Biesmeijer et al., 2006; Burkle, Marlin, & Knight, 2013; Ollerton, Erenler, Edwards, & Crockett, 2014; Potts et al., 2016). While these losses are extensively monitored in managed honeybees (Potts et al., 2010; vanEngelsdorp & Meixner, 2010), less is known on the status, trends and stressors of wild bee populations, as they are more difficult to survey (Goulson, Nicholls, Botías, & Rotheray, 2015; Potts, Biesmeijer, Bommarco, Kleijn, & Scheper, 2015). Due to the lack of adequate cost‐effective monitoring programs, trends for the vast majority of European bee species are unknown (Goulson et al., 2015; Nieto et al., 2015; Potts et al., 2010, 2016). Therefore, there is an urgent need for developing and testing comprehensive, robust and systematic monitoring programs that deliver the information needed for policymakers to decide on the most appropriate conservation measures.

To date, most monitoring programs have relied on morphological identifications, which require a sound knowledge of taxonomy and careful analysis of each individual specimen, making it a lengthy and expensive procedure (Lebuhn et al., 2013). The recent advances of “next‐generation sequencing” (NGS) techniques offer new opportunities for the assessment of biodiversity (e.g., Schnell et al., 2012; Taberlet, Bonin, Zinger, & Coissac, 2018). Molecular species identifications by DNA barcoding are particularly appealing when classical morphological identifications are not possible [e.g., eDNA, diet assessments; (Rodgers et al., 2017; Taberlet, Coissac, Pompanon, Brochmann, & Willerslev, 2012)], but DNA barcoding has also been suggested for the taxonomical assessment of morphologically identifiable taxa as a mean to reduce costs (Brunner, Fleming, & Frey, 2002; Hebert, Cywinska, Ball, & DeWaard, 2003).

Although DNA‐based monitoring methods have emerged only recently, there have been numerous efforts to establish reliable molecular identification pipelines (e.g., Gibson et al., 2015; Ji et al., 2013). For the successful implementation of NGS‐identification tools into monitoring programs, the approach should be reliable, reproducible, cost‐ and time‐effective, easily applicable and, ideally, quantitative to enable assessing species abundance (Joseph, Field, Wilcox, & Possingham, 2006). To date, a variety of tools have been developed, and even though most tools have great potential, each is associated with limitations. Presently, most approaches have been assessed in terms of accuracy (species detection and abundance), but only few have been compared with regard to costs and workload (e.g., Elbrecht, Vamos, Meissner, Aroviita, & Leese, 2017). Furthermore, substantial variation in terms of species detection rates and abundance estimates can be observed among studies applying the same molecular methods (although with slightly different parameters), casting doubt on their reproducibility (e.g., see Liu et al., 2013 and Yu et al., 2012 for interstudy variation, or Brandon‐Mong et al., 2015 for intrastudy variation). There is thus an urgent need for a comprehensive and reliable benchmark study assessing the strengths and weaknesses of different methods not only in terms of species detection and abundance estimates, but also in terms of cost and workload. In this study, we assessed and compared three NGS approaches likely to be among the most suitable to be implemented in routine monitoring programs, namely metabarcoding (MB; Taberlet et al., 2012; Yu et al., 2012), mitogenomics (MG; Zhou et al., 2013) and NGS barcoding (NGSB; Shokralla et al., 2014).

As in conventional barcoding, MB relies on the amplification of a taxonomically informative gene fragment (“barcode”). However, the DNA extraction used as template in MB comes from a bulk mixture of specimens (Ji et al., 2013), rendering quantification of species abundance difficult. With NGS methods, abundance inference is generally based on the assumption that the number of output reads correlates with the initial amount of input DNA, a proxy for biomass. Thus, if the biomass of each species in the bulk mixture was known in advance, it should theoretically be possible to infer the number of specimens per operational taxonomical unit (OTU). Nevertheless, due to the very nature of the amplification steps involved in MB, this method can be subject to heavy bias, making quantifications doubtable (Dowle, Pochon, Banks, Shearer, & Wood, 2016; Elbrecht & Leese, 2015; Elbrecht et al., 2016; Piñol, Mir, Gomez‐Polo, & Agustí, 2015; Tang et al., 2015; Yu et al., 2012).

To cope with the current lack of solid quantitative output from MB techniques, a PCR‐free approach has been suggested (Zhou et al., 2013): MG, also referred to as mitochondrial metagenomics (Crampton‐Platt et al., 2015) or mito‐metagenomics (Tang et al., 2014), an ultradeep sequencing approach using mitochondrial DNA as a “super‐DNA‐barcode” (Tang et al., 2015). Derived from bacterial metagenomics, it has been successfully applied for mitochondrial mining on arthropod communities (Choo, Crampton‐Platt, & Vogler, 2017; Crampton‐Platt et al., 2015; Gillett et al., 2014; Gomez‐Rodriguez, Crampton‐Platt, Timmermans, Baselga, & Vogler, 2015; Linard, Crampton‐Platt, Gillett, Timmermans, & Vogler, 2015; Linard et al., 2018; Liu et al., 2016; Tang et al., 2015,2014; Wilson, Brandon‐Mong, Gan, & Sing, 2019; Zhou et al., 2013). Using total DNA extraction of bulk mixtures, shotgun sequencing on high‐throughput NGS platforms is performed and raw data are bioinformatically assembled either de novo or mapped to reference databases. MG is not subject to an amplification bias, making it more suitable for quantitative inference (Gomez‐Rodriguez et al., 2015; Tang et al., 2015; Zhou et al., 2013). However, even though estimates of species abundance are approaching morphology‐based results, MG is still facing methodological limitations, mostly due to the low coverage of target sequences (Crampton‐Platt, Yu, Zhou, & Vogler, 2016). Although mitochondria are found in vast copy numbers in animals, mitochondrial DNA (mtDNA) only accounts for a small fraction of the total DNA compared to nuclear sequences. Consequently, the vast majority of data (e.g., 99.47%, in Zhou et al., 2013) produced with MG is not informative, making this approach hardly cost‐efficient. Furthermore, as initially presented, MG relies on databases containing full mitogenomes for all investigated species. Because only few full mitogenomes are currently available, this approach is not realistic at this point. To overcome this problem, Gomez‐Rodriguez et al. (2015) compared results obtained using full mitogenomic databases with those obtained using only cytochrome oxidase I (COI) reference databases and found only a slight decrease in species detection and abundance rates in the latter.

In the third method investigated here, NGSB, each specimen is processed separately from extraction to sequencing, unlike in MB and MG (Shokralla et al., 2014). Similar to MB, this method relies on the amplification of a genetic marker, but instead of amplifying from total bulk extracts, PCR amplifications are done individually. Because each specimen is uniquely tagged, this approach is quantitative by design and therefore independent of species biomass information. An additional advantage of this method is that each specimen can be preserved for subsequent identification verification or simply to be archived in natural history collections (Wang, Srivathsan, Foo, Yamane, & Meier, 2018). However, processing all specimens individually increases cost and workload related to the library preparation, which constitutes the main limitation of this approach.

To assess the suitability of these three methods for monitoring purposes, we used a data set collected under regular monitoring conditions. The data were sampled to measure the effectiveness of three different types of flower strips (FS) in promoting wild and managed bees, and the crop pollination services they provide, in Swiss agricultural landscapes. To answer this question, we compared bee species richness and abundance (relative and absolute) found across the three different types of FS. Additionally, we evaluated the influence of plant species richness on wild bee abundance and diversity.

This realistic monitoring data set allowed us to assess the performance of each NGS method with respect to variation levels found among sampling sites under realistic conditions. The number of species and specimens characterizing a data set has a large influence on the overall precision, cost and workload associated with the different NGS methods, which is why estimations of those metrics only make sense with a realistic data set. Finally, using a realistic data set allowed us to determine whether the accuracy level (presence/absence, relative and absolute abundance) of the explored methods would allow us to detect ecological patterns and reach similar conclusions, and thus validate their use in monitoring programs.

Overall, in this study we compared (a) species detection rates (presence/absence data only), (b) relative and absolute species abundances, (c) ecological patterns and finally (d) costs and workload of the three different NGS‐identification methods outlined above compared to morphological identification.

## 2 MATERIAL AND METHODS

### 2.1 Sampling

The data set (sampling material) used in this study was collected in 2017 in agricultural landscapes of the central Swiss Midland. The sampling scheme was designed to identify the effectiveness of three types of sown FS for providing foraging resources to pollinators. In total, 20 different FS were sampled three times over the flower season (two FS were collected four times and one FS two times). FS were sown either in April 2013 (FS type 1, *n* = 8), April 2016 (FS type 2, *n* = 8), and September 2016 (FS type 3, *n* = 8). All three types of FS harboured unique floral mixtures, composed of species of annual (all three types) and perennial flowering plants (types 1 and 2), which were primarily selected due to their high pollen and nectar production.

To be able to obtain quantitative information on the number of pollinators present at each sampling round, a strict sweep‐netting protocol was applied. During each sampling round, transects were slowly walked up while sweeping two times 25 sweeps with one‐minute pause in between. After 50 sweeps, the collected material was transferred into a plastic bag and directly stored at −20°C in a portable freezer.

Furthermore, during each sampling round, we monitored plant species richness, allowing us to additionally assess the importance of this parameter in promoting bees.

To determine the degree of variation within each FS, the exact same protocol was repeated within the same FS after five minutes (hereafter referred to as “transect I” and “transect II”). Transect II started from the end point of transect I. In total, the data set encompasses 122 sampling points [hereafter referred to as “communities”: (17 FS × 3 sampling rounds × 2 transects) + (2 FS × 4 sampling rounds × 2 transects) + (1 FS × 2 sampling rounds × 2 transects)].

### 2.2 Identification methods

#### 2.2.1 Morphological identification

In the laboratory, raw sampling material was sorted to isolate wild bees from plant material, other insects, as well as honeybee workers. Each specimen (*n* = 723) was then pinned, labelled, dried for at least 72 hr in a desiccator containing silica gel and identified by an expert. Most specimens were identified to species‐level, but in the following cases, morphological identifications were performed to species‐group level: *Bombus terrestris* group for workers belonging to *B. terrestris*, *B. lucorum* and *B. cryptarum*; *Halictus simplex* group for females of *H. simplex*, *H. langobardicus* and *H. eurygnathus*; and *Andrena ovatula* group for females of *A. ovatula* and *A. wilkella*. Morphological identification was complemented by Sanger sequencing using COI barcoding for all specimens identified to species‐group level and not to species level (*n* = 29) or left undetermined because of lack of intact morphological criteria (*n* = 11). For clarity, we still refer to this data set as “morphological” even if for a limited number of specimens, morphological identifications have been complemented using Sanger sequencing. Details of the Sanger sequencing protocol are given in Supporting Information S1.

#### 2.2.2 Metabarcoding

Bulk DNA extractions were performed on each community using a proteinase K solution and digested overnight at 56°C. Volumes of proteinase K solutions were adapted according to the number of specimens per community so that all specimens were immersed into the solution. To reduce costs linked to commercial kits, we purified the extracts following the Canadian Center for DNA Barcoding (CCDB) DNA extraction protocol (Ivanova, Dewaard, & Hebert, 2006). For each community, to increase species detection rates and normalize template abundance, DNA purifications were performed in triplicates and immediately pooled after extraction. To reduce workflow and limit numbers of PCRs required during the library preparation, amplification was carried out using fusion primers. In addition to the priming sequence, fusion primers have overhangs composed of Illumina indexes and a unique tag of eight base pairs (bp) designed using the software Barcode generator (Meyer & Kircher, 2010). The overhangs allow amplicons to be directly loaded onto the Illumina sequencer. To overcome the inherent limitation of Illumina platforms in sequencing low complexity libraries, we added a “heterogeneity spacer” between the labelled tag and the priming sequence, as recommended in Fadrosh et al. (2014). The PCR primer sequences of the fusion primers were those of mlCOIintF and of HCO2198 (Leray et al., 2013) and targeted a 313‐bp region of the COI gene. Overall, forward and reverse primers were 95 bp long (±3 bp). Per community, bulk amplification was performed in five different PCR replicates, each harbouring a unique combination of forward and reverse tags. Further details on MB library preparation are given in Supporting Information S2. Final library was sequenced on an Illumina MiSeq using a v3 kit (2 × 300 bp) and spiked with 20% Phix.

The majority of bioinformatics analyses (detailed in Supporting Information S3) were performed using QIIME1 (Caporaso et al., 2010). Briefly, raw data were trimmed based upon the FASTQC profile before joining paired‐end reads. After demultiplexing, adaptors, spacers and primer sequences were trimmed. Chimeric sequences were identified de novo and removed using usearch61 (Edgar, 2010). Filtered sequences were then clustered using the UCLUST algorithm (Edgar, 2010) at the default similarity threshold of 97%. Taxonomical assignment of OTUs was performed using the same algorithm by fitting reads to reference sequences. To determine the impact of database quality on the species detection performance, OTUs were assigned using two separate COI databases. The first database (“uncurated”) encompassed all available COI sequences of bee species (barcodes for ca. 2,000 species) available on BOLD (Barcode of Life Database) and GenBank (downloaded in June 2017). Additional verifications were made to ensure the presence of multiple barcodes (*n* ≥ 3) for all species present in our data set. The second database (“curated”) was downloaded from BOLD and corresponds to sequences deposited by Schmidt, Schmid‐Egger, Morinière, Haszprunar and Hebert (2015) in their extensive barcoding study on western European bees (dx.doi.org/10.5883/DS‐GBAPI). This data set was initially missing barcodes of two species present in our data set (i.e., *Andrena flavipes* and *Chelostoma florisomne*), and barcodes for these two species were downloaded from other projects on BOLD and manually added to the database. Similarly, to determine the best similarity threshold, the MB bioinformatic pipeline was run several times using different similarities thresholds (from 90% [default] to 99%). Corresponding community matrices were compared to the morphological community matrix, and the threshold performing best was retained for downstream analyses. The same empirical approach was applied to determine the optimal cross‐validation setting among replicates (i.e., minimal occurrence of a species among replicates to be validated).

#### 2.2.3 Mitogenomics

Aliquots of the DNA extracts used for MB (prior to library preparation) were sheared using an ultrasonicator (Bioruptor). The MG library was built using a commercial Illumina 96 TruSeq DNA Nano kit following the manufacturer's recommendations. To reduce differences in sequencing depth, we homogenized sequencing depth on the number of specimens per community by applying the same correction factor as for MB (Supporting Information S2). The library was sequenced on an Illumina MiSeq using a v3 kit (2 × 300 bp) and spiked with 1% Phix.

Two different bioinformatics approaches were compared [i.e., (a) de novo assembly and (b) raw read mapping], and the approach recovering the highest number of species was retained for downstream analyses. (a) The de novo assembly approach mainly followed Crampton‐Platt et al. (2015). Details are given in Supporting Information S3; briefly, libraries were quality assessed using FASTQC and residual adaptors trimmed with Trimmomatic (Bolger, Lohse, & Usadel, 2014). Then, libraries were filtered to retain only mitochondrial reads using blastn (Camacho et al., 2009) and a database containing all publically available (partial and full) mitogenomes of bee species (336 mitogenomes of 82 species; among which 18 present in our data set). Putative mtDNA reads were then assembled using IDBA‐UD (Peng, Leung, Yiu, & Chin, 2012) with a 98% similarity threshold. Contigs were mapped at a 98% similarity against a custom database using BBMap (Bushnell, 2015). Since only 18 reference mitogenomes were available for the investigated species, additional COI barcodes from the curated COI database were added to the mitogenome database. Finally, SAMtools (Li et al., 2009) was used to index and extract the number of reads that mapped reference sequences. (b) The raw read mapping approach relied on BBMap (Bushnell, 2015) to map unfiltered reads against COI reference sequences. Because only a small fraction of sequences will match to the COI reference database, it is crucial for this approach that the database is not only comprehensive, but also well curated. The presence of uncurated sequences (e.g., numts) will have a major influence upon the outcome, much more than for amplicon‐based approach where coverage‐based filtering will in most cases obliterate errors originated from the database. Therefore, only the curated database was used in this approach. To further reduce false positives due to mapping of reads in the flaking regions of COI, sequencing spanning over the classical 658‐bp COI barcoding region was filtered out of the curated database. As in Tang et al. (2015), a high similarity threshold (99%) was used to reduce false positives and reads were mapped once. Mapped reads were indexed and extracted using SAMtools (Li et al., 2009).

#### 2.2.4 NGS barcoding

Before performing bulk DNA extractions described above, a single leg of each specimen was taken for DNA extraction (one extraction per specimen) following the CCDB protocol. As for MB, fusion primers were used to amplify individually all extractions and PCRs were conducted following the same conditions as for MB. After amplification, each PCR product was examined on a 1.5% agarose gel and amplicons were pooled equimolarly as estimated based on their amplification intensity. Pooled PCR products were purified with NucleoFast 96 PCR clean‐up kits (Macherey‐Nagel) using 300 μl of PCR product per well and eluted in 100 μl ddH2O. Cleaned PCR products were sequenced on an Illumina MiSeq using a v3 kit (300 bp × 2) spiked with 20% Phix.

Data processing of the NGSB library is similar to the MB procedure. The filtered reads were clustered using UCLUST at a similarity threshold of 99%, and OTUs were taxonomically assigned using the same algorithm but with a default threshold parameter (90%). A lower taxonomical assignment threshold than for MB was used to decrease the number of unassigned OTUs since only the most abundant species assignment per specimen was retained in the final matrix. The number of false positives was therefore not affected by this lower threshold. As for MB, taxonomical assignments of OTUs were performed using the two different databases (curated and uncurated).

### 2.3 Data analyses

#### 2.3.1 Species richness

For all NGS methods, we compared species richness with morphological species richness for each community and assessed species detection rates using the Jaccard similarity index (Jaccard, 1912). To determine variation between two transects collected five minutes apart within the same FS, we also computed the Jaccard index between the samples identified based on morphology.

#### 2.3.2 Quantitative inference

In this study, species quantification (relative and absolute abundance) for both bulk methods (i.e., MB and MG) was defined as a measure of the species biomass and not numbers of specimens per species. To assess quantification accuracy for MB and MG, we correlated the number of reads per species (ln‐transformed) with the corresponding species biomass measurements. For solitary bees, dry weight can be accurately estimated by the following exponential relationship (Cane, 1987): *y *= 0.77(*x*)^0.405^, where *y* is the shortest linear distance between the wing plates (intertegular distance; mm) and *x* is the dry weight (mg). A photograph was taken of each specimen using a stereomicroscope‐mounted camera (Leica M4000), and intertegular distance was measured, which enabled to measure biomass for each specimen. To compare quantitative data on the number of specimens per species among all methods, we transformed the morphological absolute abundance (number of specimens per species) into relative abundance of biomass.

#### 2.3.3 Comparison of ecological patterns

To determine whether the detected ecological patterns would be similar across our three NGS approaches as well as the classic morphological approach, we applied the same statistical analyses on presence/absence data and on relative and absolute abundance data. First, to explore how much of the observed variance in species composition across sampling sites was explained by the identification method, we performed a nonparametric multivariate analysis of variance using distance matrices [i.e., PERMANOVA; (Anderson, 2001)]. The same test was also performed on the morphological data set to determine the biological variance found between the two transects sampled five minutes apart. These PERMANOVA tests (*adonis* function in the R cran vegan package) were performed using the Jaccard dissimilarity index for presence/absence data and the Bray–Curtis distance dissimilarity index for both relative and absolute abundance data. All *adonis* analyses were run with 10,000 permutations. Second, to complement the *adonis* analyses, we performed nonmetric multidimensional scaling (NMDS) to visualize and compare community compositions of FS among the identification methods. The goodness of fit between the superimposed shapes of NMDS plots was assessed by Procrustes tests computed with the *protest* function (vegan package). The NMDS analyses were performed with the *metaMDS* function implemented in the vegan package with the *noshare* function activated to use extended dissimilarities when sampling sites did not share species. “Spider” diagrams were added to connect communities sharing the same FS type. Third, to determine and compare the effectiveness of the three different types of FS in promoting wild bees, we ran linear mixed models (LMM) and generalized linear mixed models (GLMM) using the lme4 package (Bates, Mächler, Bolker, & Walker, 2015). Species richness and species abundance (relative and absolute) were used as response variables (see details of models in Supporting Information S12). Finally, to determine the importance of flower richness on promoting wild bees, we applied similar models with the predictor variable being the interaction between plant species richness and identification method. The relationship between plant species richness and bee richness or abundance was plotted using linear regressions with 95% confidence intervals.

### 2.4 Cost and workload

Costs estimates are based upon suppliers' prices applied in 2018 in Switzerland and do not contain cost linked to workload. To compensate for the cost of wet laboratory consumables, overall costs were increased by 15%. For the morphological identifications, the workload includes mounting, labelling and databasing of the specimens and the cost corresponds to the identifications performed by the taxonomist. Regarding the workload estimate for NGS methods, only hands‐on laboratory processes were recorded, leaving out time needed for overnight digestions, PCR amplifications, electrophoresis or other incubation times.

To predict the relationship between overall cost and total number of specimens, we divided the price per specimen into fixed (i.e., independent from the number of specimens) and variable costs (dependent on the number of specimens). For the three NGS methods, we thus subtracted the cost of the sequencing kit (variable cost) to the grand total and divided the result by the number of specimens (fixed cost). Cost estimates for morphological identifications only included fixed costs.

Finally, since Illumina platforms offer the possibility to run different kits harbouring variable outputs, we estimated the overall cost and sequencing depth for all kits allowing to span our targeted read length (~ 450 bp; including tags and technical sequences) for MB and NGSB, namely the MiSeq v3 (2 × 300 bp), MiSeq v2 (2 × 250 bp) and the MiSeq v2 Nano (2 × 250 bp) kit; and for MG, the MiSeq v3 (2 × 300 bp), HiSeq 4000 (1 × 50 bp) and HiSeq 4000 (2 × 75) kit.

## 3 RESULTS

### 3.1 Morphological identification

Wild bees were found in 83 of the 122 sampling points. After sorting wild bees from the honeybees (*n* = 1,422 honeybees) and other arthropods (mainly aphids, dipterans and coleopterans), we counted 723 wild bee specimens. A total of 683 specimens were identified morphologically to species level, 29 to species‐group level (among which 20 were identified as workers from the *B. terrestris* group), and 11 remained unidentified. Sanger sequencing, used as complement for the identification to the species level of the species‐groups and undetermined specimens, was successful for 39 of 40 specimens. The one unidentified specimen for whom Sanger sequencing failed was classified as “unidentified”.

The morphological data set, complemented with Sanger sequencing, comprised 723 specimens and 58 species, of which 382 specimens belonged to the transects I and 341 to transects II (Supporting Information S4). The median number of specimens per community was 5 and the mean (±* SD*) number 8.71 (± 10.12), with a minimum of 1 and a maximum of 55 specimens.

### 3.2 Sequencing outputs

The MiSeq runs produced 13.8, 17.5 and 9.0 million reads, respectively, for the MB, MG and NGSB libraries (Supporting Information S5). After read merging, demultiplexing and data filtering, the MB and NGSB data sets encompassed respectively 4.5 and 3.4 million reads. Raw reads from the MG library were not filtered but directly mapped to the COI reference database. In total, 28.26%, 0.02% and 32.22% of reads mapped to the database, for MB, MG and NGSB, respectively. To estimate the average coverage per specimen and community, the number of mapped reads was divided by either the number of specimens (*n* = 723) or the number of communities (*n* = 83). On average, the number of reads per specimen was 5,450, 4 and 3,959 for MB, MG and NGSB, respectively, and 47,471, 38, 34,485 per community, respectively.

### 3.3 Impact of the quality of the COI reference databases in MB and NGSB

For both MB and NGSB, species detection rates were higher while using the uncurated COI reference database (Supporting Information S6). The use of this database uncovered more true positives and decreased the number of false negatives. For NGSB, using the uncurated database, however, introduced one supplementary false positive. Based on these results, the uncurated database was used for all subsequent analyses.

### 3.4 MB parameters

Similarity thresholds for the taxonomical assignment of OTUs considerably influenced the overall number of false positives and negatives (Supporting Information S7A). The similarity threshold providing the highest species detection rates (Jaccard similarity index) was 97% and 98%. Since species detection rates were similar for 98% and 97%, the more widely accepted threshold of 97% was favoured and used in all subsequent analyses. At this threshold, the mean percentage of unassigned OTUs per community was 18.1% (Supporting Information S8).

Cross‐validation thresholds had a lesser effect and produced similar number of false positives and false negatives when validating species present in at least 1, 2, 3, 4 or 5 out of 5 replicates (Supporting Information S7B.). The less stringent thresholds (i.e., 1/5 and 2/5) introduced one additional false positive while the correlation between biomass and read numbers was slightly higher than for the more stringent thresholds. Because the higher correlation between biomass and read number did not reduce the overall difference found between the morphological and MB matrices, and because this less stringent threshold slightly increased the false‐positive rate, the more conservative threshold of three out of five replicates was favoured and used for subsequent analyses.

### 3.5 MG pipelines

The Jaccard index for the de novo assembly pipeline was considerably lower than for the raw mapping pipeline (Supporting Information S9). The former pipeline uncovered 17 true positives whereas the latter 53 true positives. Based on these results, the raw mapping pipeline was favoured for downstream analyses.

### 3.6 Species richness

The Jaccard similarity index between morphological and NGS data sets was highest for NGSB, followed by MB and MG (Table 1). For NGSB, all species present in the morphological data set were recovered and only two additional species (false positives) were identified (Supporting Information S4). The number of false negatives was similar for MB (*n* = 5) and MG (*n* = 5), although MG harboured substantially more false positives (*n* = 16) than MB (*n* = 4). There was no clear overlap in species identity between the false positives and negatives found in those two methods (Supporting Information S4). The lowest Jaccard similarity index was found among transects of the morphological identification method (Jaccard index = 0.508). Jaccard indexes between transects of each NGS method were consistently close (Supporting Information S10).

**Table 1:** Jaccard similarity index between the global diversity of morphological (Morpho) and molecular (MB, MG and NGSB) data sets. Similarity indexes per transect for the molecular methods are given in Supporting Information S10

Data sets	Transects	Species richness	# Shared species	False positives	False negatives	Jaccard index
Between transects of Morpho	I	43	30 (30/43 = 69.8%)	—	—	0.508
	II	46	30 (30/46 = 65.2%)	—	—	0.508
	I and II	58	—	—	—	—
Between MB and Morpho	I and II	57	53 (53/57 = 93.0%)	4 (4/57 = 7.0%)	5 (5/57 = 8.8%)	0.855
Between MG and Morpho	I and II	69	53 (76.8%)	16 (23.2%)	5 (7.5%)	0.716
Between NGSB and Morpho	I and II	60	58 (96.7%)	2 (3.4%)	0 (0%)	0.967

### 3.7 Quantitative inference

**Figure 1:**
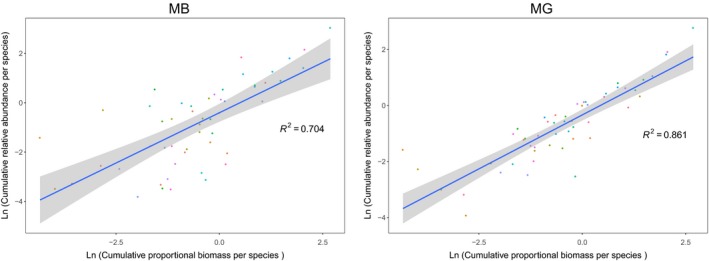
Correlation between the ln‐transformed relative read number per bee species and the ln‐transformed estimate proportional biomass per species for metabarcoding and mitogenomics data sets. Grey areas represent the 95% confidence interval. Proportions were cumulated across all sampling sites. Each coloured dot represents a different species. Correlations were significant with *p‐*values < 0.0001 [Colour figure can be viewed at http://www.wileyonlinelibrary.com]

Individual species biomass (as computed based on morphological identifications and measured intertegular distances) was significantly correlated with the sequencing output for both MB and MG for relative and absolute abundance (Figure 1, Supporting Information S11). For both NGS methods, correlations were higher when using relative abundance than absolute abundance. MG displayed higher correlation coefficients than MB, especially for relative abundance (Figure 1).

### 3.8 Ecological patterns

PERMANOVA tests, performed to analyse and quantify differences in community compositions between NGS and morphological data sets, revealed significant differences in the abundance data for both MB and MG, but not for NGSB (Table 2). With presence/absence data, the differences were significant only for MG data sets. Overall, the identification method explained 0.1%, 9.0% and 10.7% of the variance found compared to the morphological data set for MPS, MB and MG, respectively.

**Table 2:** Nonparametric multivariate analysis of variance on distance matrices (PERMANOVA) using the *adonis* function and Procrustes test (*protest* function) between NMDS of molecular (MB, MG and NGSB) and morphological (Morpho) identifications. Jaccard dissimilarity index was used to transform the presence/absence data sets and the Bray–Curtis index for both abundance formats. For the morphological identified data set, the PERMANOVA test was performed between transects

Method	Test	Levels	Presence/absence				Relative abundance				Absolute abundance			
			*df*	*F* model	*R* ^2^	*p* Value	*df*	*F* model	*R* ^2^	*p* Value	*df*	*F* model	*R* ^2^	*p* value
Morpho	PERMANOVA	Transect	1	0.729	0.009	0.796	1	0.617	0.008	0.850	1	0.617	0.008	0.852
		Residuals	80		0.991		80		0.992		80		0.992	
		Total	81		1.000		81		1.000		81		1.000	
MB	PERMANOVA	Identification	1	0.760	0.005	0.727	1	3.614	0.022	**<0.001**	1	15.809	0.088	**<0.001**
		Residuals	164		0.995		164		0.978		164		0.912	
		Total	165		1.000		165		1.000		165		1.000	
	Procrustes				0.803	**0.001**			0.819	**0.001**			0.783	**0.001**
MG	PERMANOVA	Identification	1	19.044	0.106	**<0.001**	1	10.974	0.064	**<0.001**	1	15.625	0.089	**<0.001**
		Residuals	160		0.894		160		0.936		160		0.911	
		Total	161		1.000		161		1.000		161		1.000	
	Procrustes				0.543	**0.001**			0.651	**0.001**			0.350	**0.001**
NGSB	PERMANOVA	Identification	1	0.207	0.001	1.000	1	0.251	0.001	0.995	1	0.228	0.001	0.998
		Residuals	164		0.999		164		0.999		164		0.999	
		Total	165		1.000		165		1.000		165		1.000	
	Procrustes				0.934	**0.001**			0.854	**0.001**			0.900	**0.001**

Note

*p*‐Values under the 0.05 threshold are in bold.

**Figure 2:**
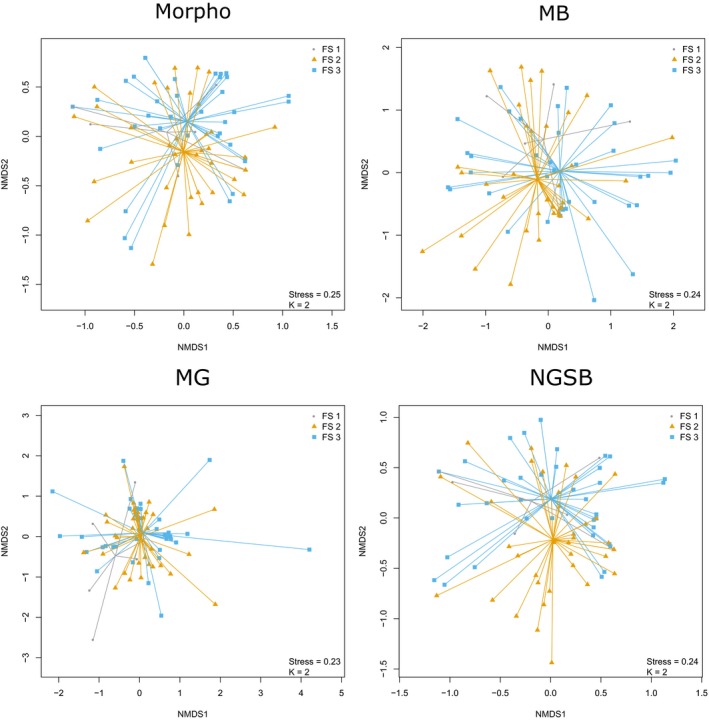
Nonmetric multidimensional scaling (NMDS) of bees' relative abundance obtained by four different species identification methods. The NMDS analyses were performed using the Bray–Curtis index with the *metaMDS* function implemented in the vegan package. “Spider” diagrams connect communities sharing the same flower stripes (FS) type. Goodness of fit between the superimposed shapes of the molecular NMDS plots with the corresponding morphological NMDS plots was assessed using Procrustes tests, computed with the *protest* function (vegan package) (see Table 2). Note the close similarity between data sets based on morphology and NGBS [Colour figure can be viewed at http://www.wileyonlinelibrary.com]

The NMDS ordinations showed similarities in community composition across the morphological and the NGS methods (Figure 2, Supporting Information S12). This was especially true for the NGSB data sets for whom the Procrustes tests revealed highly similar community compositions to the morphological one (Table 2). For the MB and MG data sets, Procrustes tests also depicted significant correlations with the morphological data set in community composition, although with lower correlation coefficients. As in PERMANOVA analyses, the lowest correlation coefficient for MB and MG was found with absolute abundance data.

**Figure 3:**
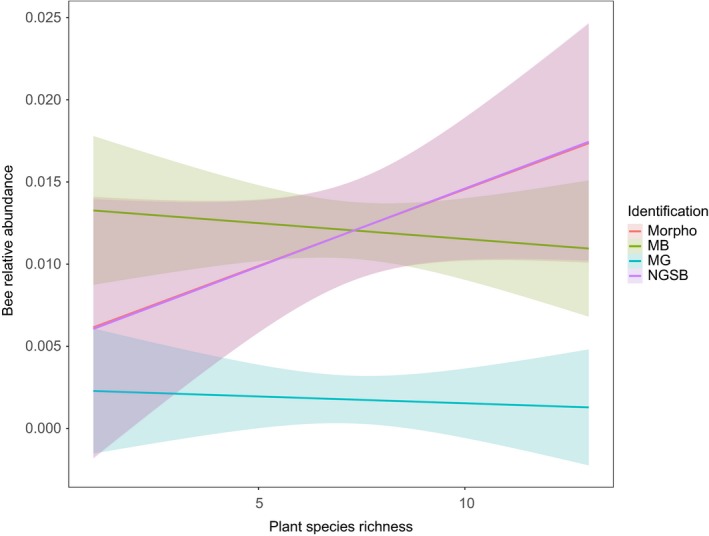
Relationship between plant species richness and the relative abundance of bees for different identification methods. Lines were computed by linear regressions as implemented in ggplot2. Coloured areas represent the 95% confidence interval. Statistical differences in relationships of the molecular identification method compared to the morphological identification method were assessed by linear mixed models. For bee species richness, no difference in relationship was found between the morphology and NGSB (regressions overlap), while MB and MG showed significant deviation compared to the relationship based on morphological identifications (See Supporting Information S15 for LMM results) [Colour figure can be viewed at http://www.wileyonlinelibrary.com]

**Figure 4:**
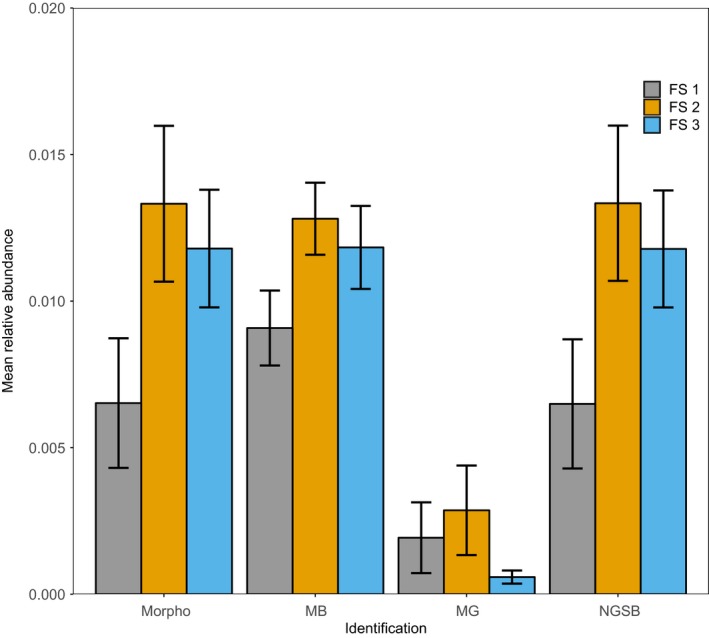
Mean relative abundance of bees for three different types of flowering strips (FS). Means were computed per identification methods, and error bars correspond to the mean standard error. Statistical difference among means within each identification method was assessed with linear mixed models. No statistical difference among types of FS was found within method [Colour figure can be viewed at http://www.wileyonlinelibrary.com]

While testing for difference in bee species richness or abundance among the three different types of FS, the GLMM (presence/absence) and LMM (relative and absolute abundance) analyses depicted no statistical difference among FS types for all identification methods (Figure 4, Supporting Information S13). Similarly, using the plant species richness as predictor, all identification methods showed comparable relationships between plant species richness and bee species richness (Supporting Information S14–S15). However, the relationships between plant species richness and bee relative abundance were significantly different from the morphological data set for MB or MG (Figure 3, Supporting Information S15). Indeed, MB and MG showed a negative relationship between bee abundance and plant species richness whereas this relationship was positive for the morphological and NGSB data sets. Furthermore, MG overall underestimated the bee relative abundance, whereas MB overestimated it for plots low in species abundance and underestimated it for species‐rich abundant plots (Figure 3).

### 3.9 Cost and workload

With respect to cost, morphological identification was approximately half the price of the cheapest NGS‐identification method (MB) and approximately three times cheaper than the priciest one (MG) (Supporting Information S16), when cost was estimated based on the number of specimens included in this study. For all investigated NGS methods, the sequencing kits used in this study represented the principal fraction of the overall cost. Since the sequencing kit cost is independent from the number of specimens sequenced (as long as the desired sequencing depth is reached), we calculated costs with increasing number of specimens. Based on this calculation, after approximately 1,795 and 4,639 specimens, MB and NGSB would become respectively more cost‐efficient than morphology‐based identifications for the MiSeq v3 kits (Supporting Information S17, see Supporting Information S18 for cost details). Because of sequencing depth limitations, MG stayed largely costlier than morphological identification. Alternatively, instead of increasing specimen numbers, cost could be reduced by using smaller, less expensive sequencing kits. Based on the mean sequencing depth of MB and NGSB, we estimated the coverage and overall cost for two alternative kits (Miseq v2 [2 × 250 bp] and MiSeq v2 Nano [2 × 250 bp]; Supporting Information S19). Although sequencing depth attained in this study for MB and NGSB was slightly underoptimal (Supporting Information S4), coverage estimations based on these figures suggest sufficient sequencing depth, even for the smallest sequencing kits (Supporting Information S19).

Regarding workload, MB was the identification method requiring less workload. Morphological and MG required similar workloads and NGSB moderately more (Supporting Information S15).

## 4 DISCUSSION

Overall, our results show that (a) NGSB provided the data set most similar to the morphological data set, both in terms of species detection and abundance. (b) As predicted, the correlation between biomass and read numbers was stronger for MG than for MB. Nevertheless, MG produced more false positives (23.2% against 7.0% for MB) and therefore considerably decreased similarities in community compositions compared to the morphological data set. (c) For both MB and MG, species abundance estimates were better when using relative abundance than absolute abundance. (d) Ecological patterns were similar across all identification methods when using presence/absence data. However, when using abundance data (both relative and absolute), the conclusions based on MB and MG identification, but not NGSB, differed from those based on morphology; (e) finally, the overall cost of all three NGS methods was higher than morphological identifications. However, MB and NGSB become more cost‐effective by either using smaller sequencing kits (e.g., MiSeq v2 Nano kit) or by increasing specimen numbers. Hereafter, we summarize the advantages and weaknesses of each NGS method.

### 4.1 Metabarcoding

Since Taberlet et al. (2012) proposed MB as a modern tool for assessing biodiversity, MB has been widely accepted when alternative means of species identification are lacking (e.g., eDNA, diet analyses). However, for cases where morphological identification is possible (e.g., pollinators surveys), MB is still in a validation phase. To date, the vast majority of MB studies have been tested against laboratory‐assembled communities of known composition (e.g., Elbrecht & Leese, 2015; Elbrecht et al., 2016; Piñol et al., 2015; Tang et al., 2015; Yu et al., 2012), and the reported detection rates are highly variable. For instance, Tang et al. (2015) compared the accuracy of MB and MG on a data set taxonomically similar to ours (33 wild bee species represented by 250 specimens) and found as many as 11 false negatives and 49 false positives, for 53 true positives. Based on these figures, the Jaccard similarity index between morphological and MB identification would be 0.47.

As illustrated in the study by Tang et al. (2015), MB detection rates are frequently obliterated by high numbers of false positives and negatives (Gentile Francesco Ficetola, Taberlet, & Coissac, 2016), a problem that strongly biases the overall interpretation of species detectability (Lahoz‐Monfort, Guillera‐Arroita, & Tingley, 2016). To overcome this limitation, replicates are crucial (Mata et al., 2019). Although it is possible to estimate the number of required replicates (Ficetola et al., 2015), the optimal replication level largely depends on the data set. In our study, we empirically tested different settings and observed no major differences among them. Although detection rates may vary across studies, to our knowledge, all rates of species detection were under 100%. Because a perfect match between NGS and morphological identification is illusive, Ji et al. (2013) investigated the effect of such discrepancies on policymaking and management issues. To do so, they compared MB with standard morphology‐based data sets and found that both exhibited similar alpha‐ and beta‐diversities, leading to similar policy conclusions. Although insightful and pioneering, this study was conducted on a very large data set (55,813 arthropods and bird specimens) in which small variations in species presence/absence would be unlikely to have a strong influence. Applying a similar approach to our much smaller data set resulted in similar conclusions: morphological and MB data sets exhibited similar species composition (Table 2), revealing similar ecological patterns with (a) no differences in bee species richness among the three different types of FS and (b) similar positive relationships between plant species richness and bee species richness (Supporting Information S14–S15).

Nevertheless, these conclusions are based on presence/absence data while the majority of monitoring programs rely on species abundance data, which gives a more precise picture of community composition (Joseph et al., 2006; MacKenzie, 2005). Therefore, there has been numerous efforts to foster the reliability of MB species count, and currently, there is an equal number of studies claiming or disclaiming quantification reliability (see Piñol, Senar, & Symondson, 2019). A study investigating the variability in quantification recorded the level of variance in read numbers associated with individual nematodes between PCR/library replicates and found an overall very consistent read count per individual (*R* ^2^ = 0.99) (Porazinska, Sung, Giblin‐Davis, & Thomas, 2010). However, their results also highlighted consistent variance in read numbers among species, even after correcting for their body size. In a similar attempt to uncover variation sources in read quantification at the interspecies level, Elbrecht and Leese (2015) sequenced libraries build with the exact same biomass of different species and found substantial differences in read abundance among species (up to four times higher or lower read abundances). These results underline an inherent problem linked to PCR‐based techniques, that is the primers' species‐specific efficiency. PCR amplification efficiency is primarily (73%) influenced by the number of template‐primer mismatches (Piñol et al., 2015), and therefore, the selection of primers will greatly influence the quantitative output (Piñol et al., 2019). While testing 15 common universal COI primer pairs, Piñol et al. (2019) found a significant relationship between DNA concentration pre‐ and post‐PCR for the vast majority of primers (14/15) although *R* ^2^ values were variable. The primer pair used in our study performed relatively well, even though other primers performed better (e.g., ArF5 & ArR5, Gibson et al., 2014). The problems outlined above likely contribute to the large differences in quantification inference reported in the literature. Furthermore, bulk‐based approaches might inform on the biomass, but not necessarily on specimen numbers because of intraspecific biomass variations (e.g., sex or “cast” polymorphism in social bees).

In our study, we found strong correlations between read numbers and estimated biomass, especially when using relative abundance data (up to *R* ^2^ = 0.704; Figure 1). The beta‐diversity of MB and morphological data sets was also highly similar for relative abundance with only 2.2% variance explained by the identification methods alone (Table 2). Furthermore, the Procrustes test depicted a relatively high correlation between the NMDS shapes of the MB and morphological data sets (*R* ^2^ = 0.819, *p*‐value < 0.001; Figure 2). Although these results are promising, we still found evidence of a bias introduced because of quantitative inference. Indeed, the LMM analysis depicted contrasting relationships between plant species richness and bee relative abundance depending on the identification method (Figure 3, Supporting Information S15); while the relationship between these variables was positive for the morphological data set, it was slightly negative for the MB data set (Figure 3). These results show that regardless of high correlations between estimated biomass and inferred abundance in morphology and MB, the overall ecological patterns are skewed by a biased estimate of species abundance, ultimately leading to incorrect ecological conclusions.

### 4.2 Mitogenomics

As initially suggested by Zhou et al. (2013) and several follow‐up studies (Gomez‐Rodriguez et al., 2015; Tang et al., 2015), we corroborate that quantitative inference based on biomass is less biased with a PCR‐free approach: regardless of the quantitative community format, Pearson's correlations were higher for MG (relative abundance: *R* ^2^ = 0.861; absolute abundance: *R* ^2^ = 0.623) than MB (relative abundance: *R* ^2^ = 0.704; absolute abundance: *R* ^2^ = 0.549) (Figure 1, Supporting Information S11). Interestingly, the correlation coefficients found in our study are similar to those found in other MG analyses (Zhou et al., 2013: *R* ^2^ = 0.64; Gomez‐Rodriguez et al., 2015: *R* ^2^ = 0.69; but see Tang et al., 2015: *R* ^2^ = 0.25).

While estimates of biomass appear to be more reliable and precise when using MG, the higher number of false positives and negatives (Table 1) skewed the overall species composition and introduced greater variance than with MB (Table 2). Although often claimed as less prone to false positives and negatives than PCR‐based methods (Tang et al., 2015; Zhou et al., 2013), we nevertheless found in our study substantially more false positives (23.2%) than with MB (7.0%). We argue that these high rates could mainly be attributed to two factors: the reference database and the low coverage. First, the database used in our study featured sequences for considerably more species (>450 species) than present in our data set (58 species). This approach was favoured to mimic monitoring conditions with limited a prior knowledge on species richness. To date, previous studies often opted for a more conservative approach and used the same DNA extracts for building the reference databases and the NGS library (e.g., Gomez‐Rodriguez et al., 2015), which most likely increases the mapping success. Additionally, using a full mitogenomes reference database has been shown to slightly decrease the false negatives and positives rates (Gomez‐Rodriguez et al., 2015), but is presently illusive for monitoring purposes due to the lack of published and annotated mitogenomes. In our study, the reduced number of false positives found with the de novo assembly approach (Supporting Information S9) also indicates that an exhaustive database can considerably improve the outcome of MG. Second, higher coverage rates could help reducing false discovery rates by filtering out all mappings under a certain threshold or by adding replicates to cross‐validate species presence/absence as we did here on the MB data set. In general, sequencing depth is a major limitation for MG as the vast majority of sequences produced with MG do not correspond to mitochondrial sequences and are therefore currently uninformative (although see Linard et al., 2015). In our study, approximately 0.02% of all reads mapped to the COI reference database for the raw read mapping pipeline (Supporting Information S5). For the de novo assembly pipeline, approximately 5% of the reads were mapped to the mtDNA reference database. Using full‐mitogenome databases unsurprisingly increases the overall percentage of mapped reads, but in most cases, the mitochondrial fraction will nevertheless plateau around 1% (see review on MG by Crampton‐Platt et al., 2016).

Despite these limitations, this PCR‐free method has the advantage of not relying on taxon‐specific primers and is therefore universally applicable to any group of animal, or even to plants, fungi or bacteria if other organelles or genes are targeted.

### 4.3 NGS barcoding

In terms of species detection and abundance, NGSB performed best by far. Indeed, we found highly similar community compositions compared to the morphological identification data (Tables 2, Figures 2, 3, 4, Supporting Information S4–S10). Noteworthy, in transect II, two specimens belonging two *H. simplex* (as determined by Sanger sequencing) were most probably miss‐identified as *H. langobardicus* by NGSB, a species for which barcoding is often challenged due to the co‐amplification of nuclear copies of mitochondrial genes (i.e., numts; unpublished data C. Praz). For most western European bee fauna, COI barcoding is reliable and provides enough resolution to discriminate at the species level; however, there are some known cases of barcode sharing. In our data set, only one problematic case of barcoding sharing species was sampled (i.e., *Andrena dorsata,* which shares barcodes with *A. propinqua*). After verification, this species was correctly identified for two out of the three methods (i.e., MB and NGSB). For MG, *A. dorsata* was not identified however neither was its sister species (i.e., *A. propinqua*). Therefore, potential biases due to barcoding sharing can be excluded in our study.

The PERMANOVA and Procrustes tests on relative or absolute abundance data also indicate high similarity between this method and morphology in terms of species abundance and ecological patterns (Figures 2, 3). The level of accuracy found in this study is in the range of previous studies. For instance, Shokralla et al. (2015) applied NGSB to a diverse data set of arthropods (11 orders) and obtained an overall recovery rate of 97.3% (*n* = 1,010), and 96.5% for Hymenoptera alone (*n* = 226). Likewise, Wang et al. (2018) sequenced over 4,000 ants using NGSB and obtained 95% of correspondence between taxonomy and morphology.

Besides high accuracy, NGSB holds several other advantages over bulk‐based approaches (i.e., MB and MG). First, individual DNA extractions and the preservation of associated specimens provide the possibility of verifying unexpected records (e.g., rare species or species outside their known range) through morphology since exoskeletons remain mostly unaltered after proteinase K digestions. Alternatively, DNA extractions can be performed on single legs as done in our study, and reference specimens could be kept nearly intact, although at the cost of additional workload. The preservation of reference specimens provides a valuable back‐up, and therefore, NGSB data are more likely to be considered for national or international databases, which can be used for purposes other than monitoring (e.g., compiling red lists or more generally for conservation biology). Second, DNA barcodes generated using NGSB can be fed into existing DNA databases since a link to the specimen is maintained. Third, DNA extractions can further be used for population genetic or phylogenetic studies. Finally, contrary to MB, NGSB does not require PCR replicates. Thereby, the sequencing runs of NGSB can encompass larger data sets and provide higher coverages and thus further reduce costs.

Dealing at the specimen instead of community level has, however, a major drawback. Individual extractions and PCRs considerably increase cost and workload linked to the library preparation. This additional workload and cost difference with bulk‐based approaches will, however, largely depend on the number of specimens sampled per community.

### 4.4 Cost and workload effectiveness

One of the main arguments brought forward for promoting NGS‐identification tools in monitoring programs is the potential cost reduction in identifications. Although often stated as more cost‐efficient than morphological identification, only few studies have systematically assessed the financial advantages of NGS tools over morphology using “real” monitoring data sets. Overall, we found that all investigated NGS‐identification methods were costlier than morphological identification (Supporting Information S16). For MB and NGSB, sequencing kits constituted the largest fraction of the total cost (Supporting Information S18). To reduce the overall cost for both methods, it is possible to either use smaller sequencing kits or increase the number of specimens by sequencing run. Based upon estimations, the smallest MiSeq sequencing kit able to span our targeted fragment would considerably decrease costs without compromising sequencing depths (Supporting Information S19). Although the output of a MiSeq v2 Nano kit (2 × 250 bp) corresponds to approximately 1/30 of a MiSeq v3 kit, the estimated coverage will remain high, with over 100 mapped reads per specimens. Higher coverages can be expected if clustering optima during sequencing runs are reached. Using the MiSeq v2 Nano sequencing kit, the overall cost of MB and NGSB is largely reduced and drop in the range of morphological identification (Supporting Information S19). Alternatively, with the same sequencing kit used in this study, we estimated that MB and NGSB become more cost‐efficient than morphology after 1,675 and 4,434 specimens, respectively. Noteworthy, several steps of our pipeline could be optimized to even further reduce cost and labour time. For instance, one could reduce hands‐on time required for DNA extraction to only a few minutes by using quick DNA extraction kits such as QuickExtract DNA Extraction kit (Lucigen; see Kranzfelder, Ekrem, & Stur, 2016). Studies reducing as much as possible laboratory costs report that sequencing can be performed for approximately 0.50$ per specimen (Wang et al., 2018). Nevertheless, such cost reduction often implies fine‐tuning protocols for the targeted taxon, mainly because DNA is amplified through direct PCR (Wong, Tay, Puniamoorthy, Balke, Cranston & Meier, 2014). Additionally, such price optimization requires running libraries on partial kits/lanes, which is not always possible or proposed by sequencing suppliers. For MG, our cost estimations on the Illumina MiSeq platform show that this method will hardly overpass morphology in terms of cost‐efficiency. Indeed, sequencing depth is a main bottleneck for this method since only a minor fraction of the data is informative. Therefore, we would recommend sequencing MG libraries on more appropriated platforms, such as HiSeq 4000, HiSeq X or even NovaSeq 6000. Although there are indications that the ability to sequence shorter fragments negatively affects the overall mitochondrial proportion, and therefore, the fraction of reads corresponding to mitochondrial DNA may be reduced on a HiSeq sequencer (Crampton‐Platt et al., 2016; Maddock et al., 2016), using larger scale sequencing platforms will drastically reduce costs and increase species detection rates.

In terms of workload, MB was the least labour‐intensive method with approximately 27% less hands‐on work than morphological identification. NGSB is unsurprisingly the method requiring most workload, although it is in a close range to MG and morphological identification. Compared to MB, NGSB relies on individual DNA extraction, which is a time demanding procedure, especially since extractions were performed on single legs. With a well‐organized protocol, the sorting and DNA extractions required for NGSB may considerably be reduced, potentially to a similar level than MB. Indeed, bulk‐based approaches such as MB or MG also require presorting of raw sampling material to isolate bees from plant material, from numerous honeybees (*n* = 1,422, thus nearly twice as many wild bees in our data set) and other insects. If none‐targeted taxa, and especially honeybees, are not removed, the sequencing depth, and therefore detection rates and biomass estimations would largely be affected.

### 4.5 Conclusions

For routine monitoring of wild bees using molecular identification methods, we recommend NGSB. The reliability and accuracy levels of this method are hardly attainable with bulk‐based approaches, especially for species abundance estimation. Furthermore, this approach provides a valuable supplementary security since specimens can be re‐examined morphologically if required. NGSB is thus more likely to yield occurrence data that can be validated and integrated into national faunistic databases and thus used by bee experts and by conservation practitioners. Feeding national faunistic databases is an important by‐product of monitoring programs (e.g., in Switzerland: http://www.biodiversitymonitoring.ch/en/home.html).

## AUTHORS CONTRIBUTION

Sampling scheme was designed and conducted by D.G., M.A. and E.K. Laboratory protocols were designed and performed by M.G. and S.B. M.G. executed the bioinformatics steps and M.G., D.G., M.A. and E.K. performed the statistical analyses. A first draft of the manuscript was written by M.G., C.P. and J.F. All authors contributed to the writing of the final version of this paper.

## Data Availability

Absolute abundance matrix of all identification methods is available at Dryad (https://doi.org/10.5061/dryad.gh830j7). This repertory also contains raw sequencing files and associated metadata.
