# Supplementary material for: Evaluating next‐generation sequencing (NGS) methods for routine monitoring of wild bees: Metabarcoding, mitogenomics or NGS barcoding
Source: Mol Ecol Resour. 2019 Apr 29;19(4):847–62. doi: 10.1111/1755-0998.13013 (PMC6850489; doi:10.1111/1755-0998.13013)
Supplement: Supplementary file 1 [file MEN-19-847-s001.docx]

Supplementary Information

**Evaluating NGS methods for routine monitoring of wild bees: metabarcoding, mitogenomics or NGS barcoding**

Morgan Gueuning^1,2*^, Dominik Ganser^3,4^, Simon Blaser^1,5^, Matthias Albrecht^4^, Eva knopp^3^

Christophe Praz^2+^ & Juerg E. Frey^1+^

*^1^Agroscope, Research Group Molecular Diagnostics, Genomics and Bioinformatics, Wädenswil, Switzerland*

*^2^Institute of Biology, University of Neuchatel, Neuchatel, Switzerland*

*^3^Institute of Ecology and Evolution, University of Bern, Bern, Switzerland*

*^4^Agroecology and Environment, Agroscope, Zürich, Switzerland*

*^5^Department of Epidemiology and Public Health Swiss Tropical and Public Health Institute, University of Basel, Basel, Switzerland*

***Corresponding author**: morgan.gueuning@unine.ch

**^+^ Co-senior authorships**

**S1: Sanger sequencing protocol.**

A fragment of the COI gene was amplified by Polymerase Chain Reaction (PCR) using the primers LCO1490 and HCO2198 (Folmer, Black, Hoeh, Lutz, & Vrijenhoek, 1994). After PCR-cleanup, linear amplification was done using Big Dye Terminator v.3.1 chemistry (Applied Biosystems) and reaction cleanup was performed using DyeEX 96 kits (Qiagen). Sequences obtained from a 3130xl Genetic Analyzer (Applied Biosystems) were assembled and taxonomically identified by BLAST queries on the NCBI GenBank database using Geneious v. 10.2.3. Samples for which amplification did not work were re-amplified using the set of primers mlCOIintF / HCO2198 (Leray et al., 2013). See details of reaction below.

| **1.PCR amplification** |  |
| --- | --- |
| **Reagents** | **Volume/sample (ul)** |
| HotStarTaq DNA polymerase Mix (2x) (Qiagen) | 10 |
| Primer F (20uM) | 0.4 |
| Primer R (20uM) | 0.4 |
| H2O | 8.2 |
| DNA | 1 |
|  |  |
| Final | 20 |
|  |  |
| PCR conditions:  Lep F/ Lep R  15min 95°C // **35** cycles of 94°C for 1 min, **45**°C for 1min and 72°C for 90 s; 72 for 10 min; hold 4°C  mlCOIintF/HCO  15min 95°C // **35** cycles of 94°C for 1 min, **45**°C for 1min and 72°C for 90 s; 72 for 10 min; hold 4°C | |
| **2. Electrophoresis**  Agarose gel 1.5%  **3.Purification** |  |
| Marchery-Nagel purification plates (as recommended by supplier) | |
|  |  |
| **4. Linear amplification** |  |
| **Reagents** | **Volume/sample (ul)** |
| BigDye Terminator Mix (ThermoFisher) | 2 |
| Primer F (20uM) | 0.2 |
| H2O | 5.3 |
| DNA | [0.5;5.8] |
|  |  |
| Final | 8 |
|  |  |
| **5.Purification** |  |
| DyeEx 96 plates (Qiagen) (as recommended by supplier) |  |

**S2: Metabarcoding library preparation details.**

PCR amplification was performed in a total volume of 20 μl with 1 μl each of 10 μM of forward and reverse primer, 10 μl of HotStartTaq DNA polymerase 5 U/μl mix (Qiagen), 7 μl of ddH2O and 1 μl of bulk DNA. PCR conditions were as follows: initial activation of 15 min at 95 °C, 40 cycles of denaturation for 40 sec at 95 °C (1 °C/sec ramping), annealing for 15 sec at 45 °C (1 °C/sec ramping), one minute ramping to 60 °C (0.250 °C/sec ramping) and extension for 2 min at 72 °C (1 °C/sec ramping), followed by a final extension at 72 °C for 7 min (1 °C/sec ramping) (Frey et al., 2013). Success of PCR amplifications was verified on a 1.5% agarose gel after pooling all barcoded PCR-replicates per community. Pooled PCR products were purified using NucleoFast 96 PCR clean-up kits (Marcherey-Nagel) and eluted in 100 μl ddH2O. Purified PCR products were then quantified using a Qubit v4 (Thermofisher Scientific). To optimize sequencing depth and have higher coverages for specimens-rich communities than for specimens-poor communities, the communities’ PCR products were pooled into a final library based upon specimen-richness (number of specimens) following this correction factor:

$Pooling volume per community=ln(\frac{200}{\left[ concentration \right]}*nb individuals within community)$

Based upon the correction factor, communities were pooled and sequenced on an Illumina Miseq using a v3 kit (300 bp x 2) spiked with 20% Phix.

**S3: Bioinformatic pipelines for metabarcoding, next generation sequencing barcoding and mitogenomics.**

**Metabarcoding**

Non-assigned OTUs, likely corresponding to contaminants, nuclear pseudogenes or chimeric sequences, were filtered out. Differences in species composition, species relative abundance and species absolute abundances (read numbers) between the five PCR-replicates were visually inspected by stacked barplots:


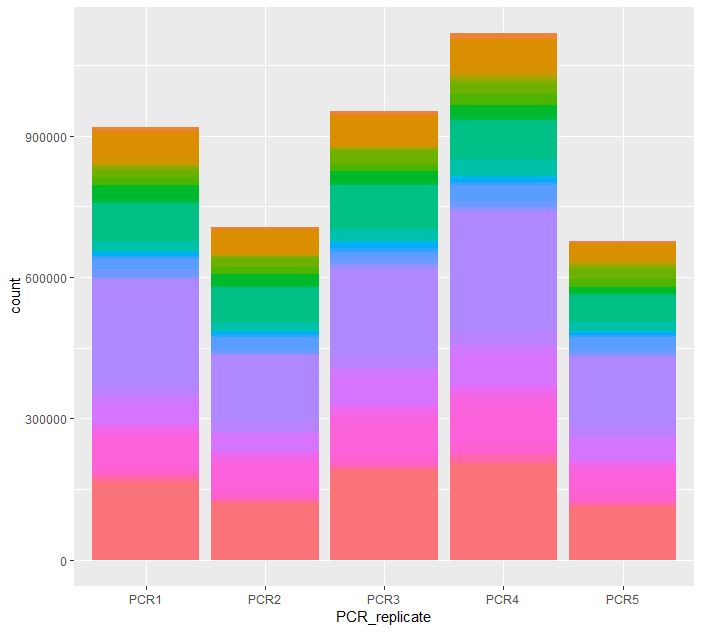


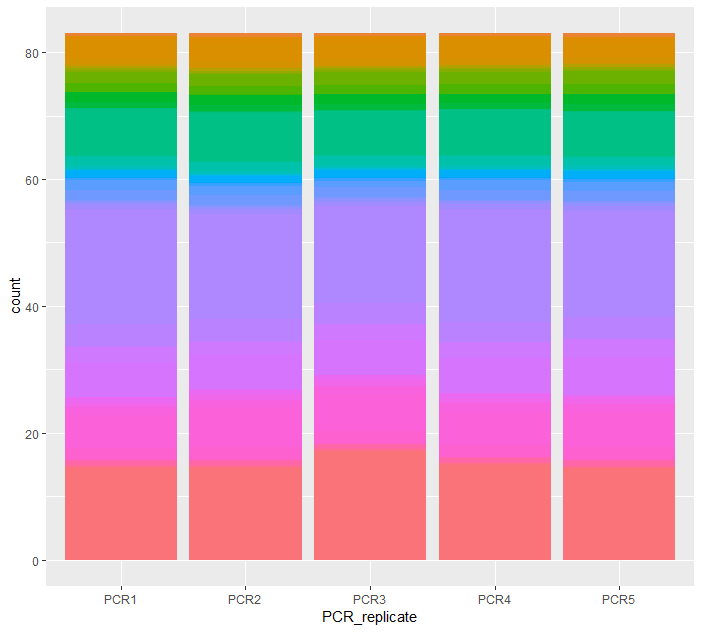


Absolute abundance

Relative abundance

*Metabarcoding abundance and relative abundance comparison in species composition between technical replicates. Each colour represents a different species.*

To reduce the number of false positives, a supplementary filter was applied to only retain taxonomical assignments that were shared among 3 different PCR-replicates per community. Read numbers were multiplied by the exponential of their pooling volume to obtain uncorrected raw abundances (see equation Supplementary Information S2). Finally, to further reduce the number of false positives, a quality filtering based on OTU abundance was applied (Bokulich et al., 2013). To find the optimal filtering threshold, we plotted decreasing numbers of species according to the filtering threshold applied:


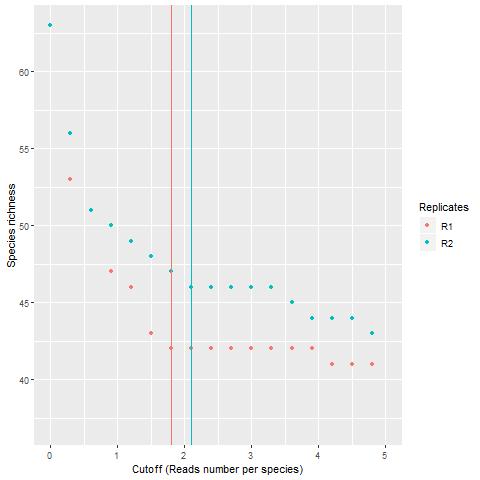


*Plot of species richness per transect according to filtering thresholds based upon read numbers.*

The optimal filtering threshold was then visually determined, similar to the process of the “elbow” method used to determine the optimal number of clusters in portioning clustering methods (e.g. K-means). Once all filtering steps were performed, the obtained absolute abundance data was transformed into relative abundance (per community) and presence/absence. Schematic workflow of bioinformatics steps is given hereunder.

**cutadapt**

**cutadapt**

Cut adaptors/spacers/primer seq

Miseq Raw data

Demultiplex

Chimeric search

Pick OTU (-s 0.97)

Filter non assigned OTU

Trim raw reads upon graph

Extract barcodes

Assign taxonomy

(-s 0.97)

Check raw data with FASTQC

Join paired end reads


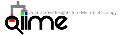

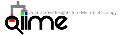

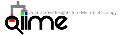

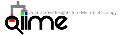


Make OTU table


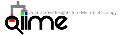

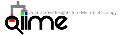

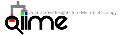


Summarize taxa

Data analyses in R


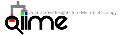

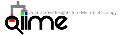

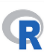


Biom table

Relative Abundance

Biom table

Absolute Abundance

**Next Generation Sequencing Barcoding**


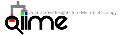

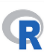


**cutadapt**

Cut adaptors/spacers/primer seq

Miseq Raw data

Demultiplex

Chimeric search

Pick OTU

(-s 0.99 with uclust)

Filter non assigned OTU

Trim raw reads upon graph

Extract barcodes

Assign taxonomy

(-s 0.90 with uclust)

Check raw data with FASTQC

Join paired end reads


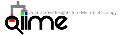

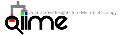

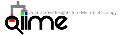

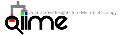


Make OTU table


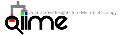

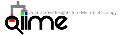

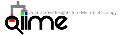


Summarize taxa

Retain most abundant taxa per sample


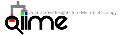

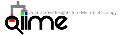


Data analyses in R

**cutadapt**

**Mitogenomics**

Check raw data with FASTQC

seq

Miseq Raw data

Filter with Trimmomatic

BBmap

(-s 0.99)

Index and extract reads

SAMtools

Data analyses in R

Blastn for mtDNA

(evalue 1e-5; max_target_seqs 1; outfmt 6)

De novo assembly

IDBA-UD (-s 0.98)

BBMap

(-s 0.98)

Index and extract reads

SAMtools

Data analyses in R

**Raw read mapping**

**De novo assembly**


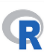

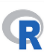


**S4: Summary table of species composition for all identification methods. Table depicts per transect the number of specimens for the morphological (Morpho) and next generation sequencing barcoding (NGSB) methods, as well as the relative abundance of read numbers for the metabarcoding (MB) and mitogenomics (MG) methods and/or the relative biomass for Morpho. To enable comparisons, the biomass of each specimen was summed and transformed into relative abundance for the morphological dataset. False negatives are highlighted in blue and false positives in red.**

|  | Transect I | | | | | Transect II | | | | |
| --- | --- | --- | --- | --- | --- | --- | --- | --- | --- | --- |
|  | Nb specimens | | Relative biomass | | | Nb specimens | | Relative biomass | | |
| Species | Morpho | NGSB | Morpho | MB | MG | Morpho | NGSB | Morpho | MB | MG |
| Andrena dorsata | - | - | - | - | 0.0001 | 1 | 1 | 0.0031 | 0.0200 | - |
| Andrena flavipes | 12 | 2 | 0.0502 | - | 0.0006 | 10 | 1 | 0.0478 | - | <0.0001 |
| Andrena fulvago | - | - | - | - | - | 1 | 1 | 0.0042 | 0.0146 | <0.0001 |
| Andrena lagopus | 3 | 3 | 0.0111 | 0.0006 | - | - | - | - | - | - |
| Andrena minutula | 2 | 2 | 0.0022 | 0.0101 | 0.0005 | - | - | - | - | - |
| Andrena ovatula | 6 | 7 | 0.0160 | 0.0669 | 0.0009 | 4 | 4 | 0.0193 | 0.0127 | <0.0001 |
| Andrena pandellei | - | - | - | - | - | 1 | 1 | 0.0045 | 0.0547 | 0.0021 |
| Andrena subopaca | - | - | - | - | - | 1 | 1 | 0.0021 | 0.0034 | <0.0001 |
| Andrena wilkella | - | - | - | - | - | 1 | 1 | 0.0063 | 0.0018 | <0.0001 |
| Bombus lucorum | 2 | 2 | 0.0331 | 0.0420 | 0.0192 | 2 | 2 | 0.0581 | 0.0142 | 0.0007 |
| Bombus subterraneus | - | - | - | - | - | 1 | 1 | 0.0266 | 0.0003 | <0.0001 |
| Bombus barbutellus | - | - | - | - | - | 1 | 1 | 0.0375 | 0.0076 | 0.0012 |
| Bombus bohemicus | 1 | 1 | 0.0155 | 0.0004 | 0.0004 | - | - | - | - | - |
| Bombus hortorum | 1 | 1 | 0.0189 | 0.0030 | <0.0001 | 2 | 3 | 0.0608 | 0.0031 | 0.0005 |
| Bombus hypnorum | - | - | - | - | - | 1 | 1 | 0.0297 | 0.0096 | 0.0004 |
| Bombus lapidarius | 3 | 3 | 0.0426 | 0.0204 | 0.0075 | 2 | 2 | 0.0369 | 0.0412 | 0.0010 |
| Bombus norvegicus | 1 | 1 | 0.0303 | 0.0014 | 0.0006 | - | - | - | - | - |
| Bombus pascuorum | 5 | 4 | 0.0740 | 0.0431 | 0.2925 | 1 | 1 | 0.0149 | 0.0007 | 0.0003 |
| Bombus pratorum | 3 | 3 | 0.0371 | 0.0362 | 0.1828 | - | - | - | - | - |
| Bombus rupestris | 1 | 1 | 0.0184 | 0.0005 | 0.0003 | - | - | - | - | - |
| Bombus sylvarum | - | - | - | - | 0.0254 | 1 | 1 | 0.0141 | 0.0040 | 0.0003 |
| Bombus terrestris | 16 | 16 | 0.2747 | 0.1685 | 0.0591 | 3 | 3 | 0.0917 | 0.1048 | 0.7834 |
| Ceratina cyanea | - | - | - | - | - | 1 | 1 | 0.0011 | 0.0024 | <0.0001 |
| Chelostoma florisomne | - | - | - | - | - | 1 | 1 | 0.0037 | 0.0088 | - |
| Chelostoma rapunculi | 5 | 5 | 0.0079 | 0.0025 | <0.0001 | - | - | - | - | - |
| Colletes similis | 3 | 3 | 0.0086 | 0.0342 | 0.0059 | - | - | - | - | - |
| Eucera nigrescens | - | - | - | - | - | 1 | 1 | 0.0179 | 0.0011 | 0.0002 |
| Halictus langobardicus | 2 | 5 | 0.0043 | 0.0321 | <0.0001 | - | 2 | - | 0.0073 | - |
| Halictus maculatus | 1 | 2 | 0.0014 | 0.0006 | <0.0001 | 1 | 1 | 0.0020 | 0.0110 | 0.0001 |
| Halictus scabiosae | 3 | 3 | 0.0110 | 0.0067 | 0.0239 | 13 | 13 | 0.0723 | 0.0143 | 0.0010 |
| Halictus simplex | 3 | 2 | 0.0068 | 0.0076 | 0.0017 | 3 | 1 | 0.0120 | 0.0189 | 0.0013 |
| Halictus subauratus | 5 | 5 | 0.0063 | 0.0123 | <0.0001 | 1 | 2 | 0.0019 | 0.0037 | - |
| Halictus tumulorum | 6 | 7 | 0.0064 | 0.0173 | <0.0001 | 12 | 12 | 0.0205 | 0.1245 | 0.0006 |
| Heriades truncorum | 4 | 4 | 0.0046 | 0.0006 | 0.0007 | 3 | 3 | 0.0074 | 0.0005 | <0.0001 |
| Hylaeus communis | 3 | 2 | 0.0049 | - | <0.0001 | 3 | 3 | 0.0033 | 0.0037 | <0.0001 |
| Hylaeus difformis | 1 | 1 | 0.0013 | 0.0050 | 0.0763 | - | - | - | - | - |
| Hylaeus gredleri | 2 | 1 | 0.0008 | - | - | - | - | - | - | - |
| Lasioglossum calceatum | 7 | 7 | 0.0155 | 0.0029 | 0.0154 | 4 | 4 | 0.0092 | 0.0003 | <0.0001 |
| Lasioglossum fulvicorne | 3 | 3 | 0.0046 | 0.0097 | 0.0001 | 2 | 3 | 0.0033 | 0.0257 | <0.0001 |
| Lasioglossum glabriusculum | 7 | 7 | 0.0035 | 0.0005 | <0.0001 | 10 | 11 | 0.0058 | 0.0010 | <0.0001 |
| Lasioglossum interruptum | 2 | 2 | 0.0029 | 0.0035 | <0.0001 | 1 | 1 | 0.0015 | 0.0039 | 0.0003 |
| Lasioglossum laticeps | 17 | 16 | 0.0218 | 0.0180 | 0.0018 | 22 | 24 | 0.0361 | 0.0236 | 0.0016 |
| Lasioglossum leucozonium | 2 | 2 | 0.0066 | 0.0032 | - | 4 | 4 | 0.0129 | 0.0020 | <0.0001 |
| Lasioglossum lineare | 4 | 4 | 0.0033 | 0.0049 | 0.0008 | 1 | 1 | 0.0012 | 0.0005 | - |
| Lasioglossum malachurum | 113 | 121 | 0.1413 | 0.2392 | 0.1069 | 81 | 87 | 0.1631 | 0.2355 | 0.0042 |
| Lasioglossum morio | 16 | 16 | 0.0104 | 0.0928 | 0.0018 | 17 | 17 | 0.0166 | 0.0450 | 0.0003 |
| Lasioglossum nigripes | 7 | 7 | 0.0175 | - | <0.0001 | 5 | 4 | 0.0189 | - | <0.0001 |
| Lasioglossum pauxillum | 43 | 39 | 0.0292 | 0.0166 | 0.0083 | 57 | 52 | 0.0601 | 0.0382 | 0.0005 |
| Lasioglossum politum | 38 | 40 | 0.0180 | 0.0295 | 0.0001 | 32 | 34 | 0.0223 | 0.1160 | 0.0003 |
| Lasioglossum puncticolle | 1 | 3 | 0.0016 | 0.0019 | - | - | - | - | - | - |
| Lasioglossum villosulum | 23 | 24 | 0.0238 | 0.0415 | 0.0002 | 22 | 21 | 0.0329 | 0.0154 | <0.0001 |
| Lasioglossum zonulum | 1 | 1 | 0.0018 | 0.0009 | <0.0001 | 2 | 2 | 0.0056 | 0.0016 | <0.0001 |
| Osmia caerulescens | 2 | 2 | 0.0081 | 0.0216 | 0.0002 | - | - | - | - | - |
| Sphecodes crassus | - | - | - | - | - | 1 | 1 | 0.0011 | 0.0003 | - |
| Sphecodes ephippius | 1 | 1 | 0.0007 | - | <0.0001 | 1 | 1 | 0.0027 | - | - |
| Sphecodes ferruginatus | - | - | - | - | - | 1 | 1 | 0.0016 | 0.0003 | <0.0001 |
| Sphecodes geoffrellus | - | - | - | - | - | 1 | 1 | 0.0013 | - | <0.0001 |
| Sphecodes puncticeps | 1 | 1 | 0.0009 | 0.0004 | <0.0001 | 4 | 4 | 0.0033 | - | - |
| Undetermined | - | - | - | - | - | 1 | - | 0.0008 | - | - |
| Andrena falsifica | - | - | - | - | - | - | - | - | 0.0005 | - |
| Andrena gravida | - | - | - | - | 0.0001 | - | - | - | - | - |
| Andrena humilis | - | - | - | - | 0.0318 | - | - | - | - | - |
| Andrena pilipes | - | - | - | - | 0.0010 | - | - | - | - | <0.0001 |
| Apis mellifera | - | 1 | - | - | 0.1203 | - | 4 | - | - | 0.1431 |
| Bombus argillaceus | - | - | - | - | 0.0021 | - | - | - | - | - |
| Bombus cryptarum | - | - | - | - | 0.0048 | - | - | - | - | 0.0281 |
| Bombus wurflenii | - | - | - | - | 0.0053 | - | - | - | - | 0.0280 |
| Dasypoda suripes | - | - | - | - | - | - | - | - | - | <0.0001 |
| Eucera interrupta | - | - | - | - | - | - | - | - | - | 0.0001 |
| Halictus compressus | - | - | - | 0.0003 | - | - | - | - | - | - |
| Halictus gavarnicus | - | - | - | - | - | - | - | - | 0.0010 | - |
| Hylaeus intermedius | - | 1 | - | - | - | - | - | - | - | - |
| Hylaeus kahri | - | - | - | - | <0.0001 | - | - | - | - | - |
| Hylaeus moricei | - | - | - | - | <0.0001 | - | - | - | - | <0.0001 |
| Hylaeus punctulatissimus | - | - | - | - | <0.0001 | - | - | - | - | - |
| Lasioglossum albipes | - | - | - | - | - | - | - | - | - | <0.0001 |
| Lasioglossum clypeare | - | - | - | - | - | - | - | - | - | <0.0001 |
| Lasioglossum crenicornis | - | - | - | 0.0005 | - | - | - | - | 0.0004 | - |
| Seladonia gavarnica | - | - | - | - | - | - | - | - | - | <0.0001 |
| Sphecodes niger | - | - | - | - | 0.0004 | - | - | - | - | - |
| Total nb Specimens | 382 |  |  |  |  | 341 |  |  |  |  |
|  |  |  |  |  |  |  |  |  |  |  |

**S5: Sequencing output and read mapping information for metabarcoding (MB), mitogenomics (MG) and next generation sequencing barcoding (NGSB) libraries. The average species and community coverage were computed by dividing the number of matching reads by the total number of specimens (n = 723) and communities (n = 83). For MG, raw reads were directly mapped to the reference database without filtering.**

|  | Total read number  (per direction) | Read number  after filtering | Read number matching  the Apoidea database | Average specimen  coverage | Average community coverage |
| --- | --- | --- | --- | --- | --- |
| MB | 13,828,724 | 4,530,461 | 3,940,098 (28%) | 5450 | 47,471 |
| MG | 17,483,469 | NA | 3126 (0.018%) | 4 | 38 |
| NGSB | 9,055,239 | 3,449,357 | 2,862,255 (32%) | 3959 | 34,485 |

**S6: Results of two different COI reference datasets on species detection for MB and NGSB. For both methods, two different reference datasets were used two assign OTU’s taxonomy. The uncurated dataset encompassed all available COI sequences of Apoidea members (barcodes for ca. 2000 species) presently available on BOLD and Genbank (downloaded in June 2017). The curated dataset encompassed sequences deposited on BOLD by Schmidt and colleagues (2015) in their barcoding study on western-Europeans bees (**[**dx.doi.org/10.5883/DS‐GBAPI**](http://dx.doi.org/10.5883/DS-GBAPI)**). Both datasets were verified to harbor barcodes for all investigated species. In case of lacking sequences, additional barcodes were download form BOLD and added manually to the databases.**

**The Jaccard similarity index were computed between the global diversity of the molecular and morphological methods.**

| Method | Database | Species Richness | # Shared species | False Positives | False Negatives | Jaccard Index |
| --- | --- | --- | --- | --- | --- | --- |
|  |  |  |  |  |  |  |
| MB | Uncurated | 57 | 53 | 4 | 5 | 0.855 |
|  | Curated | 57 | 52 | 5 | 6 | 0.825 |
| NGSB | Uncurated | 60 | 58 | 2 | 0 | 0.967 |
|  | Curated | 57 | 55 | 2 | 3 | 0.917 |

**S7: Result for different bioinformatic parameters for metabarcoding identifications. Jaccard indexes was computed based on the number of shared species between the morphological and molecular identification method. The Adonis results were computed by non-parametric multivariate analysis of variance (PERMANOVA; *Adonis* function) between morphological and metabacoding community matrixes and represent the proportion (R^2^) of variance explained by the solo effect of the identification method for presence/absence (PA), absolute abundance (AB) and relative abundance (RA) data. Asterisk (*) represent values that significantly explain de variance between both morphological and MB matrices (Significance codes: 0 ‘***’ 0.001 ‘**’ 0.01 ‘*’). Parameters that were retained for downstream analyses are marked in bold. Table A depict result for different similarity thresholds in uclust between the OTU’s representative sequence and the reference database. Table B depicts results for different cross-validation settings between replicates (i.e. minimal time a species has to occurrence between replicates to be validated) using the best similarity threshold found in table A.**

**A.**

| uclust % | Species Richness | # Shared  Species | False Positives | False Negatives | Jaccard Index | Adonis PA | Adonis AB | Adonis RA |
| --- | --- | --- | --- | --- | --- | --- | --- | --- |
| 90% | 64 | 50 | 14 | 8 | 0.694 | 0.010* | 0.091*** | 0.023*** |
| 95% | 66 | 52 | 14 | 6 | 0.722 | 0.018*** | 0.089*** | 0.026*** |
| 96% | 59 | 51 | 8 | 7 | 0.773 | 0.007 | 0.089*** | 0.023*** |
| **97%** | **57** | **53** | **4** | **5** | **0.855** | **0.005** | **0.088***** | **0.022***** |
| 98% | 57 | 53 | 4 | 5 | 0.855 | 0.008 | 0.088*** | 0.022*** |
| 99% | 31 | 30 | 1 | 28 | 0.508 | 0.041*** | 0.064*** | 0.045*** |

**B.**

| uclust % | Replicate  Filtering | Species  Richness | # Shared  Species | False  Positives | False  Negatives | Jaccard  Index | Adonis  PA | Adonis  AB | Adonis  RA | Cor_AB | Cor_RA |
| --- | --- | --- | --- | --- | --- | --- | --- | --- | --- | --- | --- |
| 97% | 1/5 | 58 | 53 | 5 | 5 | 0.841 | 0.005 | 0.088*** | 0.022*** | 0.582 | 0.745 |
| 97% | 2/5 | 58 | 53 | 5 | 5 | 0.841 | 0.005 | 0.088*** | 0.022*** | 0.582 | 0.745 |
| **97%** | **3/5** | **57** | **53** | **4** | **5** | **0.855** | **0.005** | **0.088***** | **0.022***** | **0.549** | **0.704** |
| 97% | 4/5 | 57 | 53 | 4 | 5 | 0.855 | 0.005 | 0.088*** | 0.022*** | 0.549 | 0.704 |
| 97% | 5/5 | 57 | 53 | 4 | 5 | 0.855 | 0.005 | 0.088*** | 0.022*** | 0.534 | 0.674 |

**S8: Information on number of unassigned OTU's per community. (A.) Stacked barplot of the taxonomical assignment of OTU's per family. Yellow proportions correspond to the unassigned OTU's. The plot was drawn using the QIMME v1** (**Caporaso et al., 2010**) s**cript “plot_taxa_summary.py” (B.) Summary statistics on the number of unassigned OTU's per community.**

**A.**

**
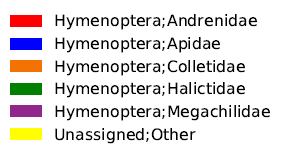
**


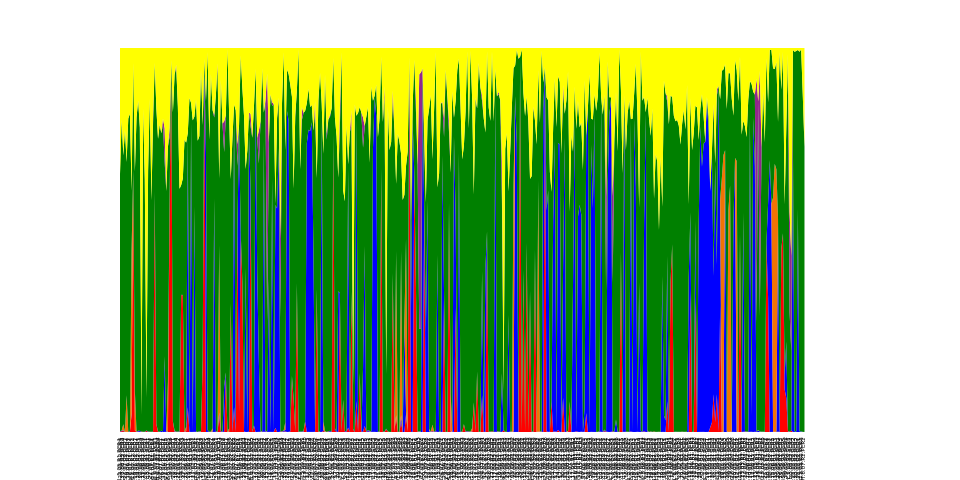


**B.**

| Min  unassigned OTU's | 1st Qu  unassigned OTU's | Median  unassigned OTU's | Mean  unassigned OTU's | 3rd Qu  unassigned OTU's | Max.  unassigned OTU's |
| --- | --- | --- | --- | --- | --- |
| 0.002485 | 0.110934 | 0.163157 | 0.181541 | 0.231936 | 0.956276 |

**S9: Jaccard similarity index between the global diversity of morphological and two different bioinformatic pipelines for the mitogenomics dataset.**

| Datasets | Methods | Transects | Species Richness | # Shared Species | False Positives | False Negatives | Jaccard Index |
| --- | --- | --- | --- | --- | --- | --- | --- |
| Between MG and Morpho | Raw read mapping | I & II | 69 | 53 | 16 | 5 | 0.716 |
|  | De novo assembly | I & II | 35 | 17 | 18 | 41 | 0.224 |

**S10: Jaccard similarity index between the global diversity of morphological and molecular datasets by transects. Percentages were computed upon the morphological species richness of the respective transect.**

| Datasets | Transects | Species Richness | # Shared Species | False Positives | False Negatives | Jaccard Index |
| --- | --- | --- | --- | --- | --- | --- |
| Between transects of Morpho | I | 43 | 30 (30/43 = 69.8%) | NA | NA | 0.508 |
|  | II | 46 | 30 (30/46 = 65.2%) | NA | NA | 0.508 |
| Between MB and Morpho | I | 40 | 38 (38/40 = 95%) | 2(2/40 = 5%) | 5 (5/40 =12.5 %) | 0.844 |
|  | II | 44 | 40 (40/44 = 90.9%) | 4(4/44 = 9.1%) | 6 (6/44 = 13.6%) | 0.800 |
| Between MG and Morpho | I | 53 | 39 (73.6%) | 14 (26.4%) | 4 (7.5%) | 0.684 |
|  | II | 48 | 38 (79.2%) | 10 (20.8%) | 8 (16.7%) | 0.678 |
| Between NGSB and Morpho | I | 45 | 43 (95.6%) | 2 (4.4%) | 0 (0%) | 0.956 |
|  | II | 47 | 45 (95.7%) | 2 (4.2%) | 1 (2.1%) | 0.937 |

**S11: Correlation between the ln transformed absolute read numbers per species and the ln transformed absolute estimate of biomass per species for the metabarcoding and mitogenomics datasets.**


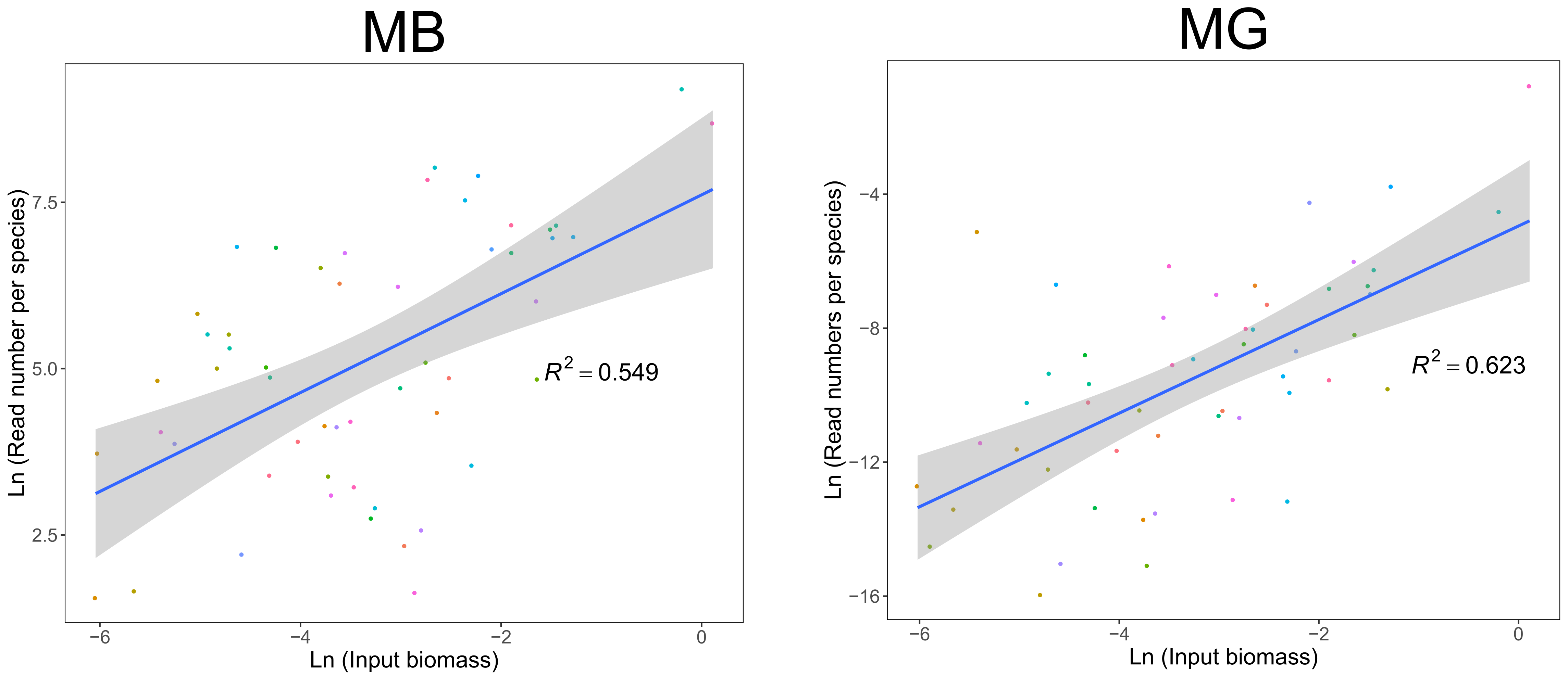


**S12: Non-metric multidimensional scaling (NMDS) on (I) occurrence dissimilarity matrices computed with the Jaccard index and (II) absolute abundance using dissimilarity matrices computed with the Bray-Curtis index. The NMDS analyses were performed with the “*metaMDS*” function implemented in the *vegan* package. “Spider” diagrams connect communities sharing the same type of flower strip (FS). Goodness-of-fit between the superimposed shapes of the molecular NMDS plots with the corresponding morphological NMDS plots were assessed using Procrustes tests computed with the “*protest*” function (*vegan* package). Results of the Procrustes tests are given in Table 2**.

I.


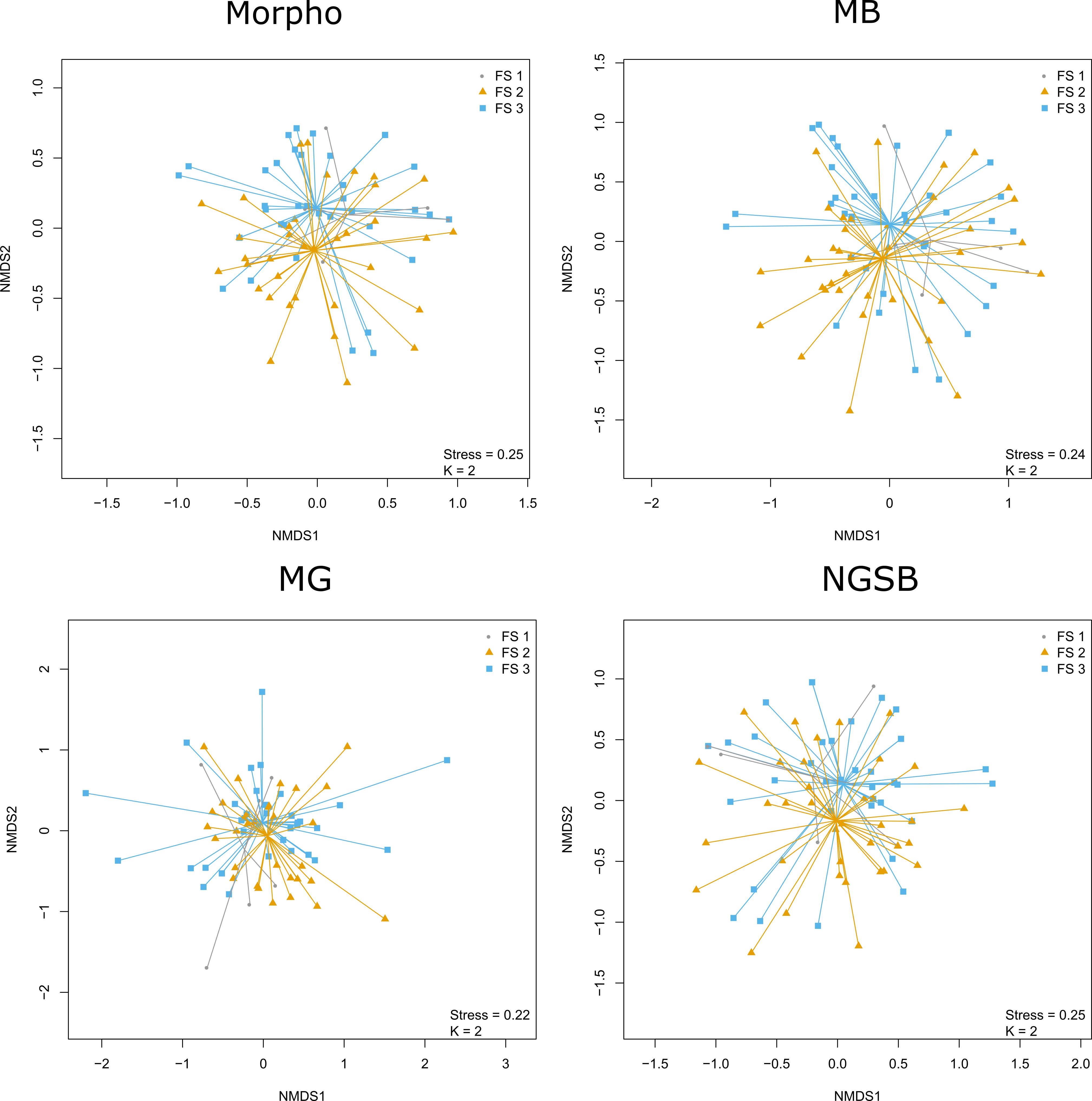


II.


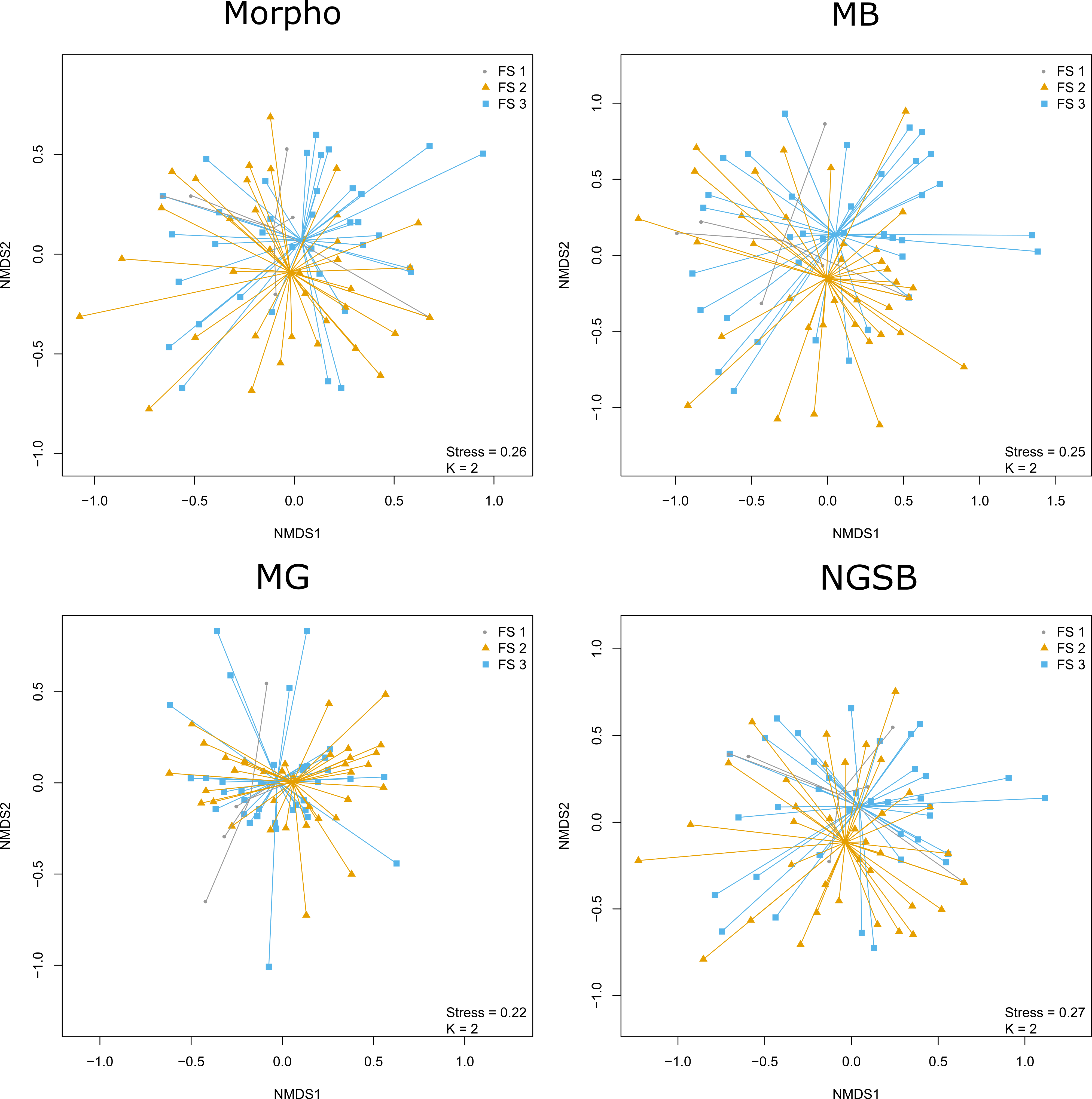


**S13: Mean bee (I) species richness and (II) absolute abundance for 3 different types of flowering strips (FS). Means were computed per identification methods and error bars correspond to the mean standard error. Statistical difference between means within each identification method was assessed by generalized linear mixed models (for species richness) or linear mixed models (for relative and absolute abundance). No statistical difference between types of FS was found within method. See manuscript for details on models.**

**Model details:**

Because we were interested in determined and comparing bee species richness and abundance between the different types of FS within each method, the predictor variable was set as the interaction between FS type and identification methods. The transects within sampling sites were used as a random factor. For species richness, we ran a GLMM using the “glmer” function and the poisson family. For abundance data, we ran LMM using the “lmer” function on both absolute and relative abundance data using the same model as above. Relative abundance was computed by dividing the sum of species (Morphological dataset and NGSB) or reads (MB and MG) for each site by the total number of species or reads per identification method. Difference in mean (± SE) species richness or abundance between FS types were graphically displayed using ggplot2 (Wilkinson, 2011).

I.


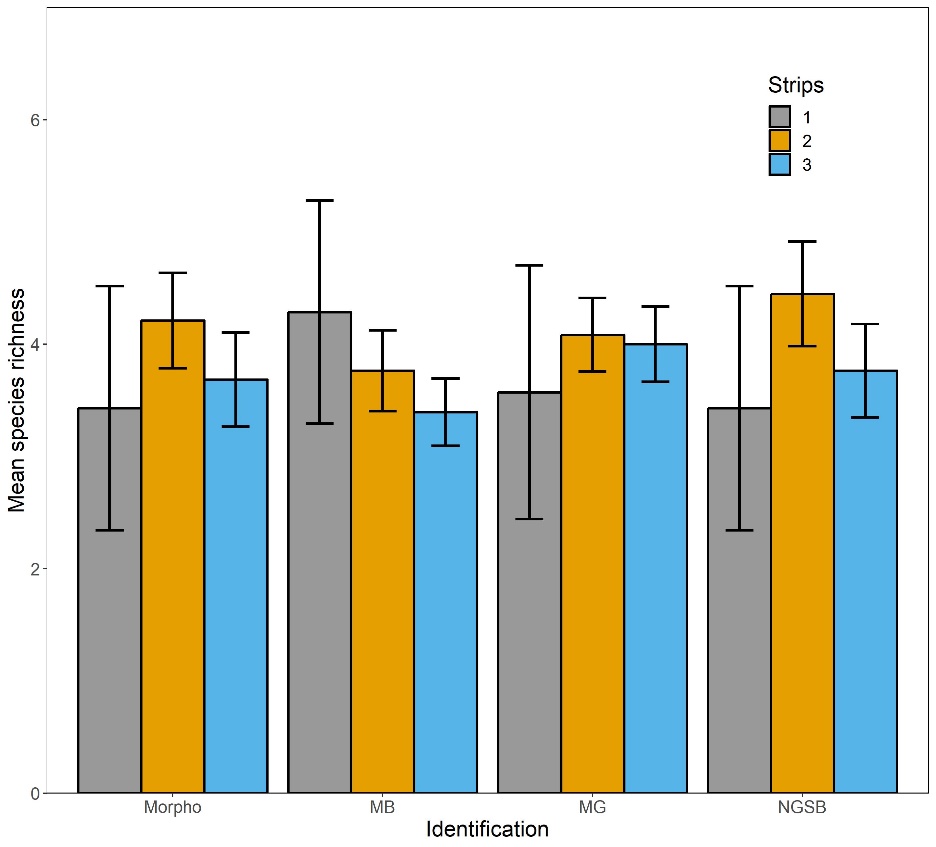


II.


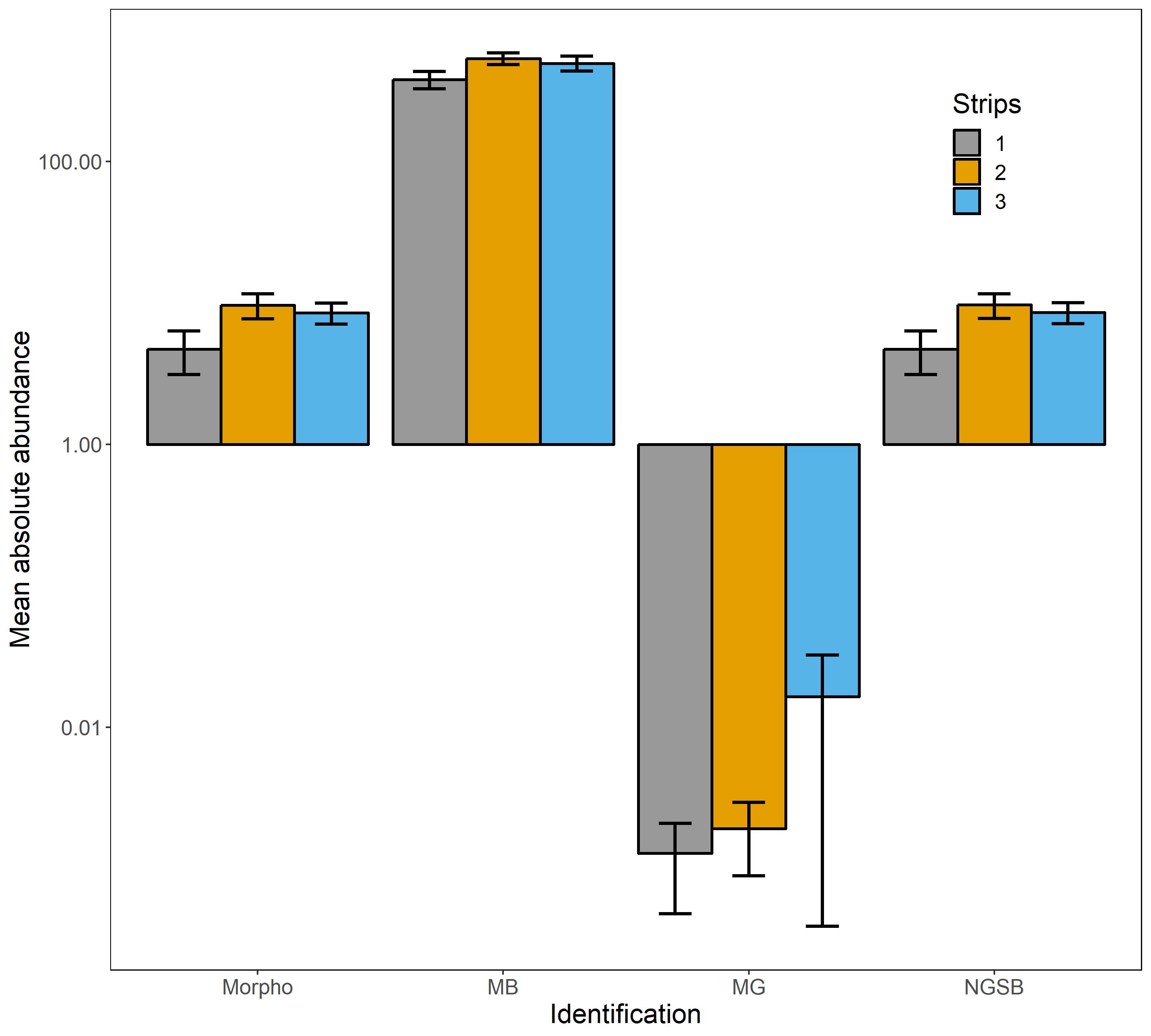


**S14: Relationship between plant species richness and (I) bee species richness or (II) bee absolute abundance different identification methods. Lines were computed by linear regressions as implemented in ggplot2. The grey areas represent the 95% confidence interval. Statistical difference between the relationship of the molecular identification method compared to the morphological identification were assessed by generalized linear mixed models (for species richness) or linear mixed models (for relative and absolute abundance). For bee species richness, no difference in relationship were found between the morphological and molecular identifications. For bee absolute abundance, MB and MG showed significant deviation to the morphological relationship towards plant species richness.**

I.


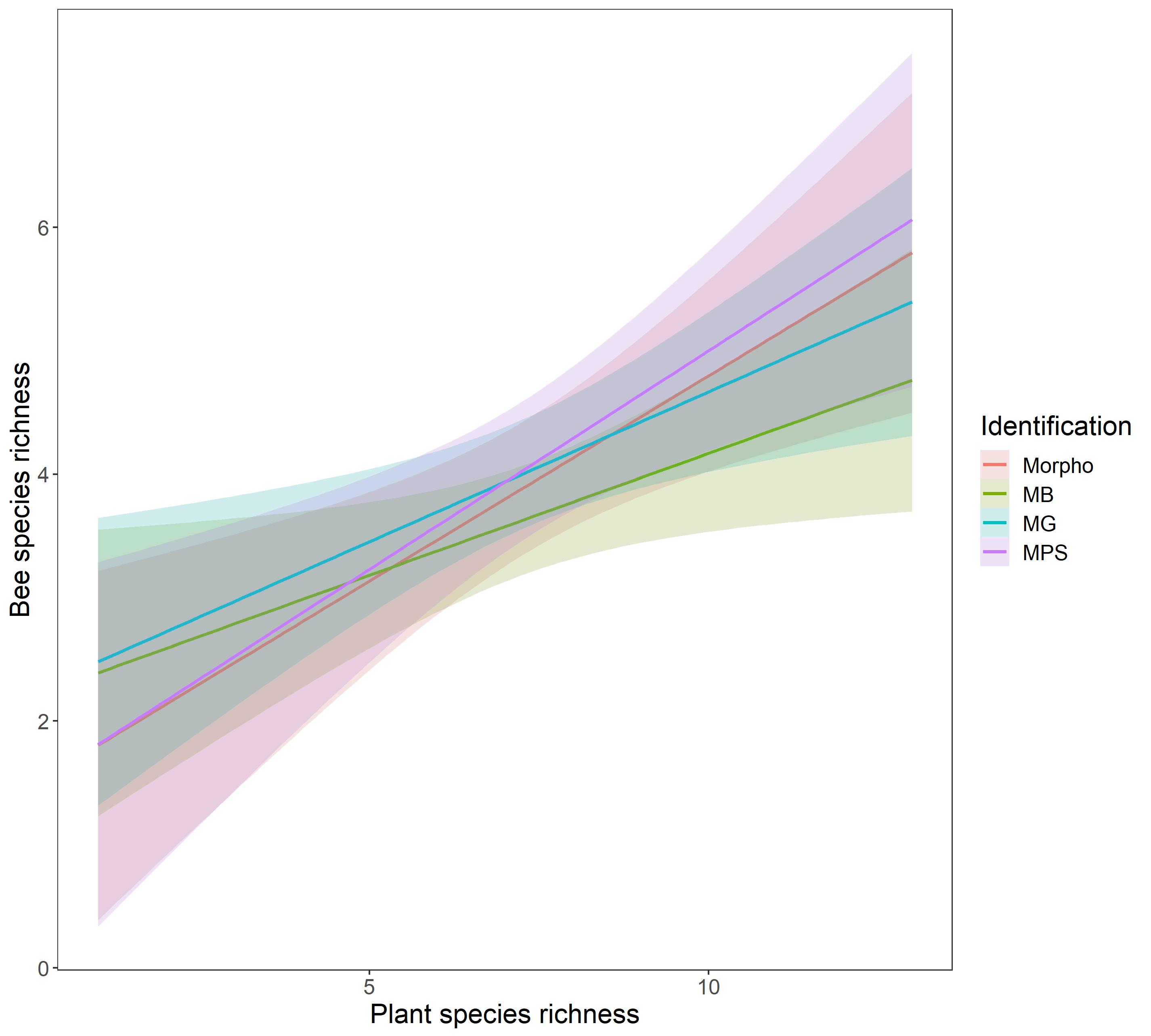


II.


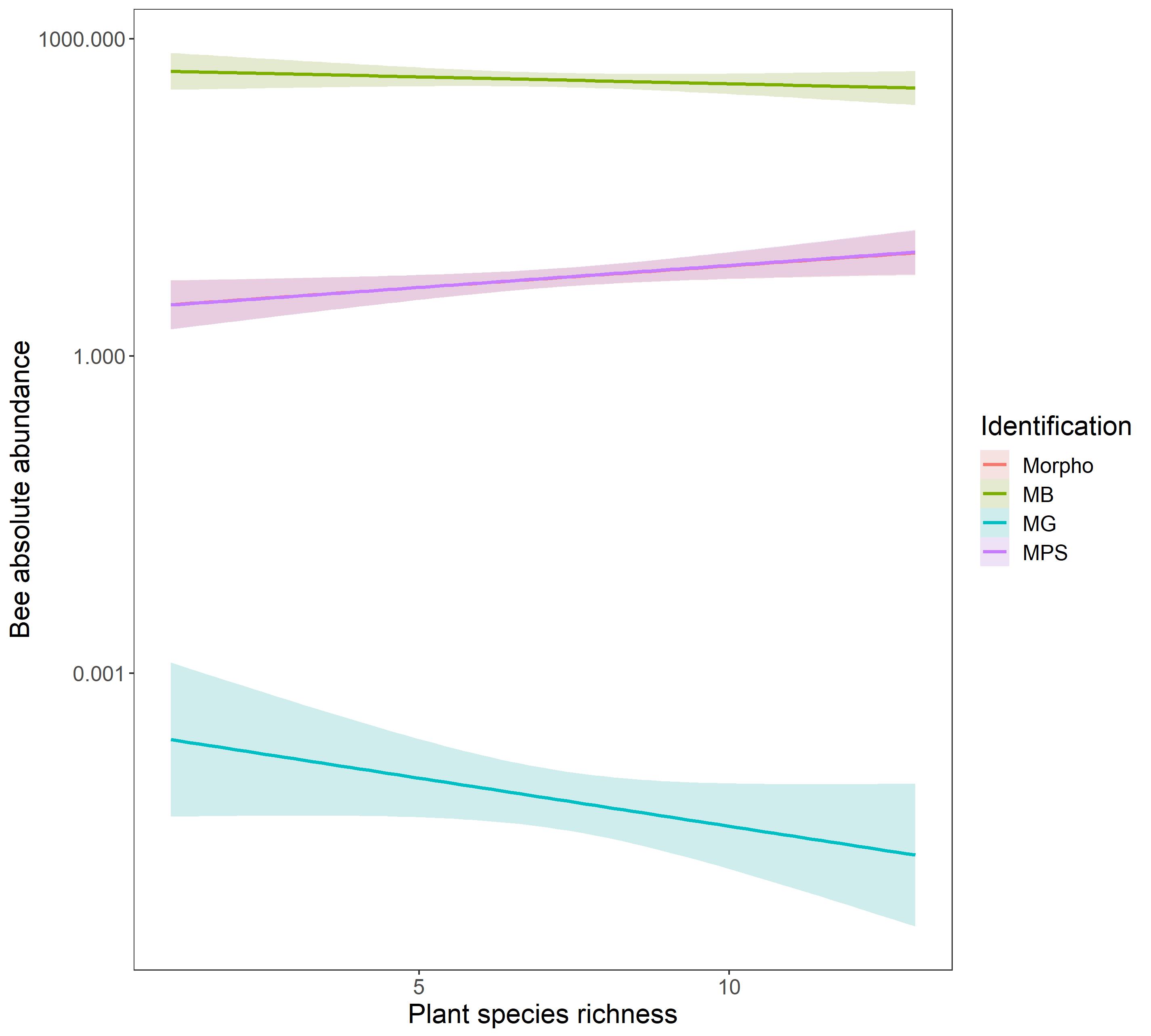


**S15: Linear mixed models (LMM) and generalized linear mixed models (GLMM) models and output between the plant species richness (plant_sr) and the bee species richness and species abundance (relative and absolute). Figures in brackets correspond to the standard error.**

Bee species richness: *m1 <-glmer(bee_sr ~ plant_sr*Identification + (1|Site/Transect), data=PA_matrix, family = poisson(link = "log"),control=glmerControl(optimizer="bobyqa", optCtrl=list(maxfun=100000)))*

Bee absolute abundance: *m2 <-lmer((log(Abs_Abundance+1)) ~ plant_sr*Identification +(1 |Site/Transect), data= AB_matrix)*

Bee relative abundance: *m3 <-lmer((sqrt(Rel_Abundance)) ~ plant_sr*Identification +(1 |Site/Transect), data= RB_matrix)*

|  | **Dependent variable**  **(estimates ± SE)** | | |  |
| --- | --- | --- | --- | --- |
|  | **Presence/Absence** | **Log(Absolute abundance+1)** | **sqrt(Relative abundance)** | |
|  |  |  |  | |
| Plant species richness (plant_sr) | 0.084** | 0.076** | 0.004* | |
|  | (0.029) | (0.028) | (0.002) | |
|  |  |  |  | |
| MB | 0.167 | 4.843*** | 0.045* | |
|  | (0.248) | (0.298) | (0.019) | |
|  |  |  |  | |
| MG | 0.225 | -1.386*** | -0.033 | |
|  | (0.247) | (0.301) | (0.020) | |
|  |  |  |  | |
| NGSB | 0.018 | -0.007 | -0.001 | |
|  | (0.245) | (0.295) | (0.019) | |
|  |  |  |  | |
| plant_sr:MB | -0.030 | -0.105** | -0.005* | |
|  | (0.030) | (0.038) | (0.002) | |
|  |  |  |  | |
| plant_sr:MG | -0.024 | -0.075 | -0.006* | |
|  | (0.030) | (0.039) | (0.003) | |
|  |  |  |  | |
| plant_sr:NGSB | 0.002 | 0.002 | 0.0001 | |
|  | (0.03) | (0.038) | (0.002) | |
|  |  |  |  | |
| Constant | 0.633** | 1.377*** | 0.067*** | |
|  | (0.233) | (0.215) | (0.014) | |
|  |  |  |  | |
| Observations | 328 | 327 | 327 | |
| Log Likelihood | -636.048 | -336.180 | 533.372 | |
| Akaike Inf.Crit. | 1292.096 | 694.360 | -1044.745 | |
| Bayesian Inf.Crit. | 1330.026 | 736.050 | -1003.055 | |
| Note: *p<0.05;**p<0.01;***p<0.001 |  |  |  | |

**S16: Cost and workload estimates. All prices are estimated upon suppliers’ prices applied for Switzerland in 2018 and are given in Swiss francs (1 CHF ~ 1 USD). Prices are exclusive of additional costs related to disposables (e.g. tips, PCR plates, insect pins and storage boxes). Estimation of total workload are based upon the hand-on time required for all laboratory and bioinformatics analyses. For the morphological identification method, workload does not encompass the taxonomist working hours, which are included into the grand total price.**

| Method | Grand total (CHF) | Price/Specimen (n = 723) | | Price/Community (n = 83) | | Workload  (hand-on) |
| --- | --- | --- | --- | --- | --- | --- |
|  |  | Excl.  consumables | Incl.  consumables | Excl.  consumables | Incl.  consumables |  |
| Morpho | 1250.- | 1.73.- | 1.80.- | 15.06.- | 15.66.- | 22:17:00 |
| MB | 2237.- | 3.09.- | 3.55.- | 26.95.- | 30.99.- | 16:12:00 |
| MG | 4340.- | 5.99.- | 6.89.- | 52.29.- | 60.13.- | 21:32:00 |
| NGSB | 2645.- | 3.65.- | 4.20.- | 31.86.- | 36.64.- | 24:32:00 |

**S17: Cost estimations per total number of specimens. Morphological identification is assumed to have a fixed price per specimen. For the three molecular identification methods, price per individual is divided by fixed (extraction, PCR, purification, etc.) and variables costs (sequencing kit). For MG, sequencing depth limit was fixed at 723 specimens and therefore each 723 specimens the cost a new sequencing kit was added. Prices are given in Swiss francs (1CHF ~ 1USD).**


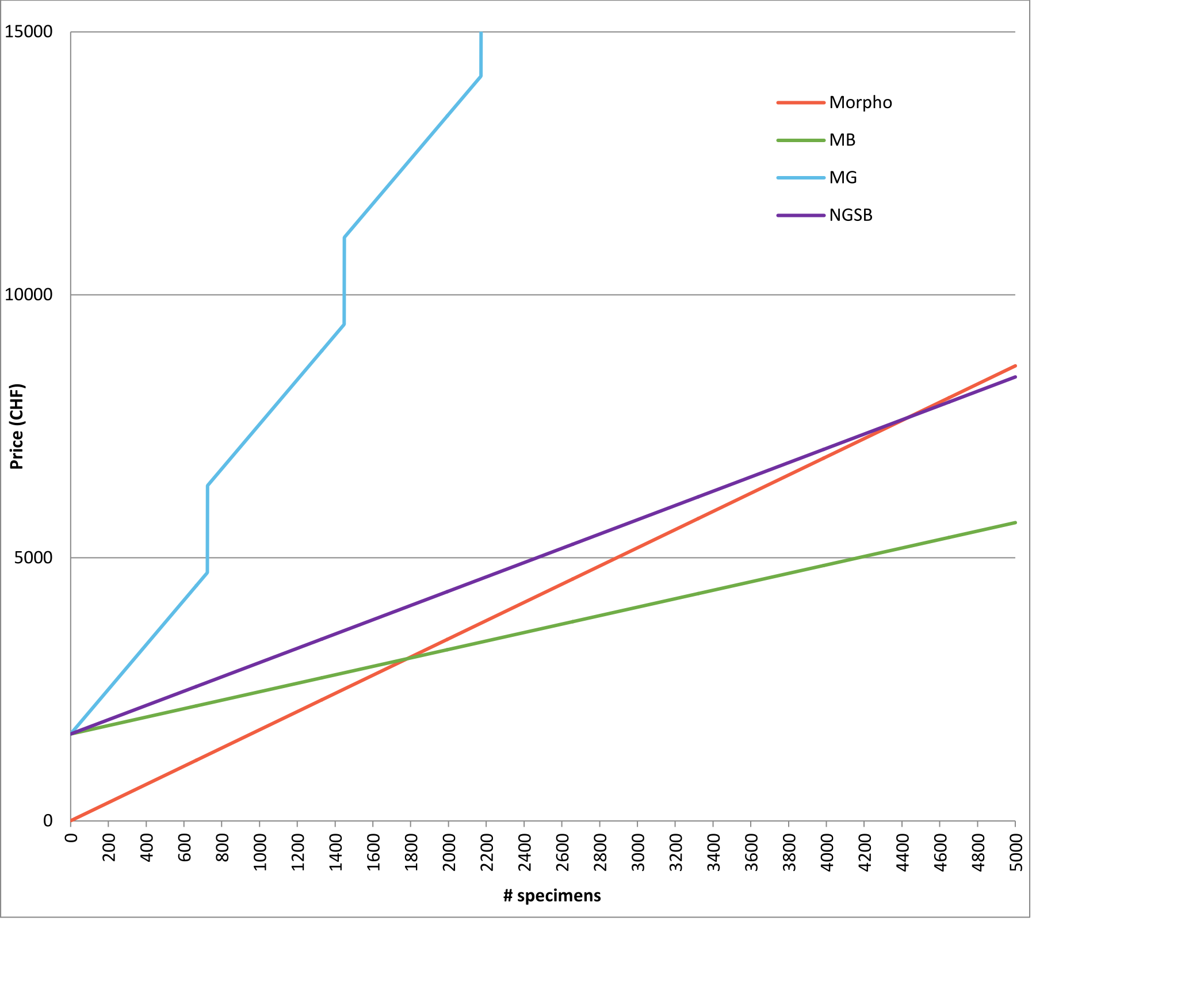


**S18: Cost details of all laboratory steps required for the library preparation and sequencing of metabarcoding (MB), mitogenomics (MG) and next generation sequencing barcoding (NGSB). Table A depicts details of bulk purchases used to compute the price per reaction. Table B shows the number of reaction used by method and the overall cost of each library step. Grand totals are given at the end of table B. All prices are given in CHF (1 CHF ~ 1 USD) and are based on the suppliers prices for the year 2018.**

| **A.** |  |  |  |  |  |  |
| --- | --- | --- | --- | --- | --- | --- |
| **Laboratory Step** | **Item** | **Company** | **Price (CHF)** | **Units** | **Volume per reaction** | **Price/reaction** |
| **DNA extraction** | AcroPrep 96 3.0 um | PALL | 250.- | 5*96 well-plates | NA | 0.521.-/ext. |
|  | Proteinase K (Lyophilized) | Promega | 116.- | 5000 ul | Variable | 0.023.-/ul |
| **PCR** | Hot StarTaq Master Mix Kit | QIAGEN | 1760.- | 25 ml (2500U) | 10 ul | 0.70.-/PCR |
|  | Genomic scale, desalted, dried primers | Microsynth | 90.- | 1700ul (100uM) | 0.08 ul/primer (100 uM) | 0.008.-/PCR |
| **Electrophoresis** | Agarose NEEO ultra-quality | ROTH | 606.6.- | 1 kg | 2.5 g/96 well-plate | 0.016.-/well |
|  | Ethidium Bromide Solution  (10 mg/ml) | BIO-RAD | 121.- | 10 ml | 25 ul/96 well-plate | 0.003.-/well |
| **PCR clean-up** | NucleoFast 96 PCR | Machery-Nagel | 2704.- | 50*96 well-plates | NA | 0.563.-/well |
| **Commercial library-prep** | 96 TruSeq DNA Nano | Illumina | 2828.- | 96 samples | NA | 29.458.-/sample |
| **Sequencing** | Miseq Reagents Kit v3  (600 cycles) | Illumina | 1650.- | NA | NA | 1650.-/run |

| **B.** | |  | |  | |  | |
| --- | --- | --- | --- | --- | --- | --- | --- |
| **Laboratory steps** | | **MB** | | **MG** | | **NGSB** | |
| **Item** | **Price/reaction** | **# reactions** | **Price** | **# reactions** | **Price** | **# reactions** | **Price** |
| AcroPrep 96 3.0 um | 0.521.-/extraction | 249 extr. (83 comm.*3 repl.) | 129.7.- | 249 extr. (83 comm.*3 repl.) | 129.7.- | 723 specimens | 376.7.- |
| Proteinase K (Lyophilized) | 0.023.-/ul | 5000ul | 115.- | 5000ul | 115.- | 3765.625 ul (5.2 ul/sample) | 86.6.- |
| Hot StarTaq Master Mix Kit | 0.70.-/PCR | 415 (83 comm.* 5 repl.) | 290.5.- | NA | - | 723 PCRs | 506.1.- |
| Genomic scale, desalted, dried primers | 0.008.-/PCR | 415 (83 comm.* 5 repl.) | 3.3.- | NA | - | 723 PCRs | 5.8.- |
| Agarose NEEO ultra-quality | 0.016.-/well | 83 samples | 1.3.- | NA | - | 723 samples | 11.6.- |
| Ethidium Bromide Solution (10 mg/ml) | 0.003.-/well | 83 samples | 0.2.- | NA | - | 723 samples | 2.2.- |
| NucleoFast 96 PCR | 0.563.-/well | 83 samples | 46.8- | NA | - | 10 wells (300 ul/well) | 5.6.- |
| 96 TruSeq DNA Nano | 29.458.-/sample | NA | - | 83 | 2445.- | NA | - |
| Miseq Reagents Kit v3 (600 cycles) | 1650.-/run | 1 | 1650.- | 1 | 1650.- | 1 | 1650.- |
| **Grand total (CHF)**  **without consumables** | | | **2237.-** |  | **4340.-** |  | **2645.-** |
| **Grand total (CHF)**  **with consumables^1^** | | | **2572.-** |  | **4991.-** |  | **3042.-** |
| ^1^ Consumables were estimated to increase cost by 15% | | | | | | | |

**S19: Estimation of cost and sequencing coverages for different Illumina sequencing kits/platforms. The output of each kit are based upon the manufacture’s figures. Mean coverage per specimens and community were compute upon the number of mapped reads using a Miseq v3 kit. Prices are given in Swiss francs (1CHF ~ 1USD), including consumables.**

| **Kit** | **Read**  **length** | **Output^1^** | **Price kit^3^** | **MB** | | | | **MG** | | | | **NGSB** | | | |
| --- | --- | --- | --- | --- | --- | --- | --- | --- | --- | --- | --- | --- | --- | --- | --- |
|  |  |  |  | **Sp.**  **coverage** | **Com.**  **coverage** | **Overall cost^2^** | **Cost/**  **specimen** | **Sp.**  **coverage** | **Com.**  **coverage** | **Overall cost^2^** | **Cost/**  **specimen** | **Sp.**  **coverage** | **Com.**  **coverage** | **Overall cost^2^** | **Cost/**  **specimen** |
| Miseq v3 | 2 x 300 bp | 13.2-15 Gb | 1650.- | 5450 | 47471 | 2572.- | 3.56 | 4 | 38 | 4991.- | 6.90 | 3959 | 34485 | 3042.- | 4.21 |
| Miseq v2 | 2 x 250 bp | 7.5-8.5 Gb | 1259.- | 3095 | 26960 | 2181 | 3.02 | - | - | - | - | 2250 | 19585 | 2651 | 3.67 |
| Miseq v2 Nano | 2 x 250 bp | 500 Mb | 359.- | 160 | 1425 | 1281 | 1.77 | - | - | - | - | 118 | 1034 | 1751 | 2.42 |
| Hiseq 4000 | 1 x 50 bp | 105-125 Gb | 817.- | - | - | - | - | 32 | 304 | 4158 | 5.75 | - | - | - | - |
| Hiseq 4000 | 2 x 75 bp | 325-375 Gb | 1193.- | - | - | - | - | 100 | 950 | 4534 | 6.27 | - | - | - | - |
| ^1^ Claimed output by Illumina.  ^2^ Cost were inflated by 15% to compensate for consumable prices  ^3^ Price for Miseq kits are based upon Illumina online shop; Price for Hiseq are based upon prices applied by NYU lagone health sequencing center (for CI member prices) and converted into Swiss francs. | | | | | | | | | | | | | | | |

|  |  |
| --- | --- |

References:

Bokulich, N. A., Subramanian, S., Faith, J. J., Gevers, D., Gordon, J. I., Knight, R., … Caporaso, J. G. (2013). Quality-filtering vastly improves diversity estimates from Illumina amplicon sequencing. *Nature Methods*, *10*(1), 57–59. doi:10.1038/nmeth.2276

Caporaso, J. G., Kuczynski, J., Stombaugh, J., Bittinger, K., Bushman, F. D., Costello, E. K., … Knight, R. (2010). QIIME allows analysis of high-throughput community sequencing data. *Nature Methods*, *7*(5), 335–336. doi:10.1038/nmeth.f.303

Folmer, O., BLACK, M., HOEH, W., Lutz, R., & Vrijenhoek, R. (1994). DNA primers for amplification of mitochondrial cytochrome c oxidase subunit I from diverse metazoan invertebrates. *Molecular Marine Biology and Biotechnology*. doi:10.1371/journal.pone.0013102

Frey, J. E., Guillén, L., Frey, B., Samietz, J., Rull, J., & Aluja, M. (2013). Developing diagnostic SNP panels for the identification of true fruit flies (Diptera: Tephritidae) within the limits of COI-based species delimitation. *BMC Evolutionary Biology*. doi:10.1186/1471-2148-13-106

Leray, M., Yang, J. Y., Meyer, C. P., Mills, S. C., Agudelo, N., Ranwez, V., … Machida, R. J. (2013). A new versatile primer set targeting a short fragment of the mitochondrial COI region for metabarcoding metazoan diversity: application for characterizing coral reef fish gut contents. *Frontiers in Zoology*, *10*(1), 34. doi:10.1186/1742-9994-10-34

Wilkinson, L. (2011). ggplot2: Elegant Graphics for Data Analysis by WICKHAM, H. *Biometrics*. doi:10.1111/j.1541-0420.2011.01616.x
